# Luminescence Thermometry 2.0: Machine-Learning Temperature Read-Out with Luminescence of YOF: Yb^3+^, Er^3+^ Nanoparticles

**DOI:** 10.3390/nano16191238

**Published:** 2026-09-30

**Authors:** Zoran Ristić, Jovana Periša, Aleksandar Ćirić, Milica Maričić, Anđela Rajčić, Tamara Gavrilović, Željka Antić, Miroslav D. Dramićanin

**Affiliations:** 1 Centre of Excellence for Photoconversion, Vinča Institute of Nuclear Sciences—National Institute of the Republic of Serbia, University of Belgrade, P.O. Box 522, 11001 Belgrade, Serbia; risticz@vin.bg.ac.rs (Z.R.); jburojevic@vinca.rs (J.P.); aleksandar.ciric@vin.bg.ac.rs (A.Ć.); msekulic@vinca.rs (M.M.); andjela.rajcic@vin.bg.ac.rs (A.R.); tamarag@vin.bg.ac.rs (T.G.); zeljka.antic@vinca.rs (Ž.A.); 2 National Institute of Research and Development for Electrochemistry and Condensed Matter, INCEMC, Str. Dr. A. Păunescu Podeanu Nr.144, 300569 Timisoara, Romania

**Keywords:** luminescence thermometry, optical thermometry, luminescence intensity ratio, Er^3+^/Yb^3+^ green upconversion, near-infrared thermometry, lanthanide-doped nanophosphors, machine learning

## Abstract

Here, we evaluate a full-spectrum, data-driven readout framework, termed Luminescence Thermometry 2.0, in which normalized emission spectra are used directly as inputs to temperature regression. We benchmark this approach against conventional luminescence-intensity ratio thermometry using the same experimental dataset obtained from the upconversion and downshifting emissions of Er^3+^,Yb^3+^-co-doped yttrium oxyfluoride nanoparticles. Relative to the best-performing conventional LIR calibration, full-spectrum regression reduces the absolute prediction bias by up to approximately 2.5-fold and the prediction spread by 24% for visible green upconversion. For near-infrared emission, the corresponding reductions reach approximately 2.2-fold and 8.6-fold, respectively. The approach reduces feature-selection bias and exploits temperature-encoded information that conventional analysis discards. Shapley additive explanations for the best-performing Gaussian process regression model identify the Er^3+^ spectral regions that dominate temperature prediction, linking the improved performance to physically meaningful emission features. These results demonstrate improved temperature readout using full-spectrum regression for YOF:Er^3+^/Yb^3+^ under the investigated conditions. Matched synthetic-background tests showed that training-set augmentation reduced background-induced set-point bias, with effects that depended on the regression method and normalization.

## 1. Introduction

Accurate temperature measurement is a cornerstone of both fundamental science and industrial engineering [1]. For several decades, luminescence thermometry (LT) has leveraged the temperature-dependent optical responses of luminescent materials. Historically, research has focused on two parallel strategies: developing new materials with high thermal sensitivity and refining techniques to extract temperature from measured signals [2,3]. Despite significant progress in material design and readout methods, a plateau in accuracy and precision has emerged, prompting exploration of different data-analysis strategies.

Most luminescence-thermometry readouts nevertheless remain based on a limited number of manually selected spectral parameters [1,4,5,6]. Following the methodological classification proposed in our recent SpectraSensML study [7], feature-based luminescence thermometry can be grouped into three categories: conventional single-feature readouts (LT1.0), enhanced single-feature approaches (LT1.1), and methods combining multiple extracted thermometric parameters (LT1.5). Full-spectrum, data-driven temperature readout is designated LT2.0. These labels distinguish how spectral information is used; they do not imply a universal ranking of measurement performance.

Conventional Single-Feature Methods (LT1.0): This foundational approach involves extracting and calibrating isolated spectral features as a function of temperature. Researchers typically select a single temperature-dependent parameter, such as the luminescence intensity ratio (LIR), luminescence lifetime, spectral line position, or bandwidth, and establish an empirical calibration relationship for temperature [6,8,9].

Enhanced Single-Feature Methods (LT1.1): To enhance sensitivity while preserving conceptual simplicity, these approaches introduce dual-probe or innovative single-parameter techniques, such as dual-activated lanthanide [10,11] or transition-metal and lanthanide co-doped phosphors [12,13], single-band ratiometric (SBR) temperature read-out [14,15,16], multilevel cascade LIR (McLIR) read-out [17], luminescence intensity squared (LIR^2^) read-out [18], and deconvolution-assisted methods [19,20]. While these approaches offer improvements, they remain incremental and perpetuate the practice of partial spectral utilization.

Multiparametric Methods (LT1.5): Recently, statistical tools, such as Multiparameter Linear Regression (MLR) and sensor fusion, have been employed to extract information from multiple parameters simultaneously [3,21,22,23,24,25,26], thereby improving thermometric performance.

All these feature-based methods are inherently biased because they rely on subjective (though physics-guided) emission-peak selection and baseline-correction procedures, which may introduce significant errors into the thermometric analysis. Beyond these methodological limitations, feature-based luminescence thermometry also faces physical barriers; for example, the sensitivity of the LIR is limited by the energy difference between emission levels, which cannot be increased indefinitely without compromising the thermalization required for accurate sensing [3].

**Figure 1:**
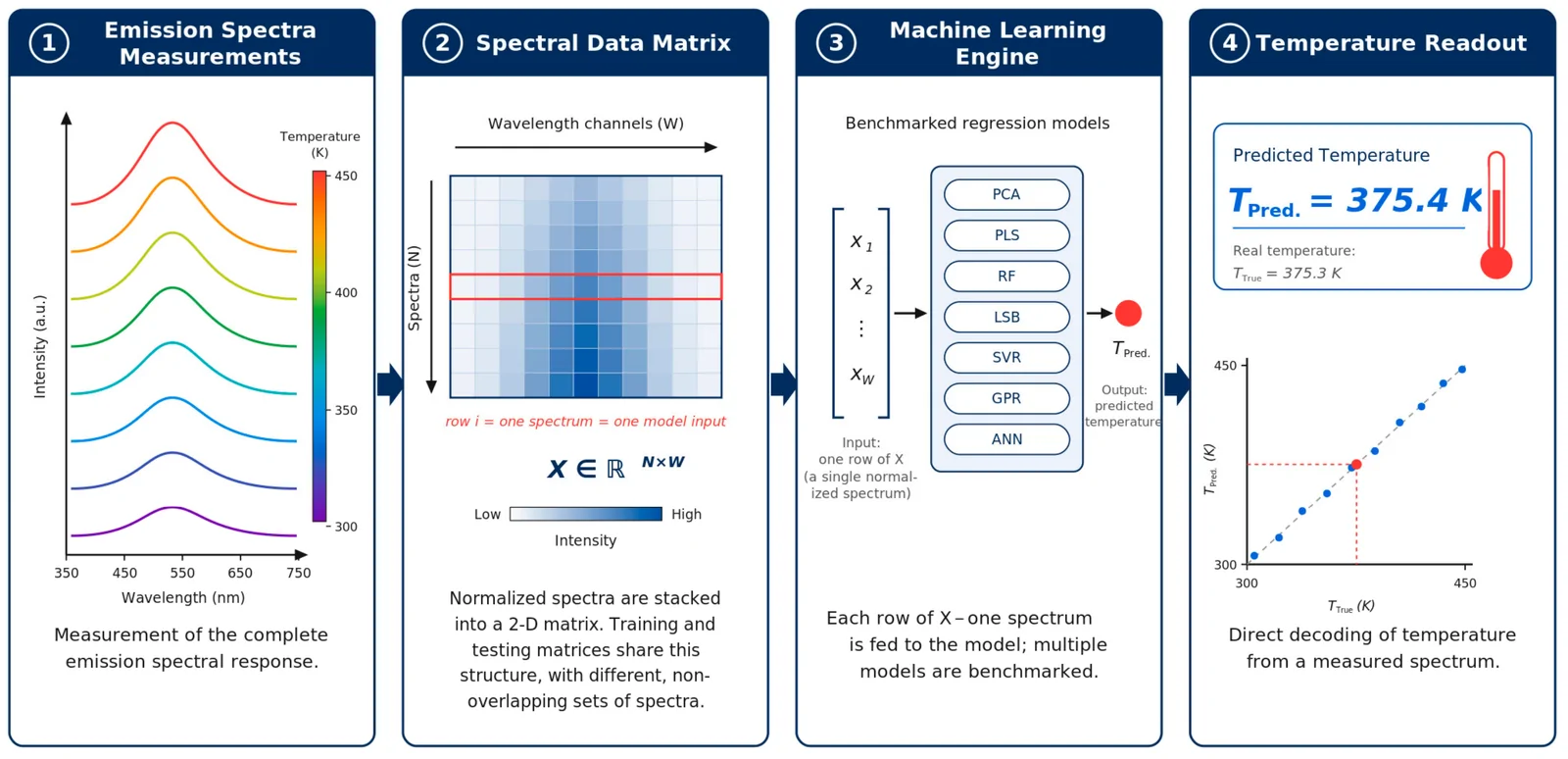
Full-spectrum machine-learning workflow for Luminescence Thermometry 2.0. The complete temperature-dependent emission spectrum is acquired rather than reducing the optical response to manually selected spectral features. Each spectrum is normalized and represented as a high-dimensional vector of wavelength-dependent emission intensities, and multiple spectra are stacked into a data array for regression modeling. An ML engine then learns the mapping between full-spectrum information and temperature, enabling direct temperature readout together with an estimate of prediction uncertainty. In contrast to conventional LIR thermometry, this approach uses the full temperature-encoded spectral response and avoids manual peak selection, baseline definition, and feature engineering.

To address these limitations and extend recent applications of machine learning in luminescence thermometry [27,28,29,30,31] and nanomaterials [32,33,34,35,36,37,38,39,40], we investigate a full-spectrum readout in which the normalized intensity at every recorded wavelength is supplied to regression models without prior selection of individual emission bands. This full-spectrum readout follows the framework termed Luminescence Thermometry 2.0 (LT2.0) in Ref. [7]. Repeated spectra acquired at stabilized calibration temperatures are normalized and arranged as an *N* × *W* matrix, where *N* is the number of spectra and *W* is the number of wavelength channels. In this study, each training dataset contained *N* = 800 spectra, comprising 50 spectra at each of 16 calibration temperatures from 300 to 450 K at 10 K intervals. The VIS and NIR datasets contained *W*_VIS_ = 175 and *W*_NIR_ = 160 wavelength channels, respectively, giving training matrices of 800 × 175 and 800 × 160. Regression models are then trained to map each spectrum to the corresponding stabilized temperature. This procedure avoids manual selection of individual emission bands and allows distributed spectral changes to contribute to temperature prediction; its performance is evaluated using spectra excluded from model development. Its workflow is conceptually elegant and significantly simpler to implement than conventional luminescence thermometry (see Figure 1). The machine learning (ML) engine uses all temperature-relevant information to build a regression model that maps high-dimensional spectroscopic data to temperature values. Since ML engines process the full emission spectrum, they can access all temperature-relevant information that conventional LT systematically discards and integrate these disparate signals into a temperature readout with presumably much better precision and accuracy than traditional methods provide.

Here, we test this full-spectrum LT concept using Er^3+^/Yb^3+^-co-doped YOF nanoparticles, for which established synthesis procedures and characterized upconversion and downshifting luminescence provide a suitable platform for comparing thermometric readouts. A particular advantage is that the same material provides two complementary test cases: visible green upconversion emission from the thermally coupled Er^3+ 2^H_11/2_ and ^4^S_3/2_ levels, which constitutes a widely used benchmark for LIR thermometry, and near-infrared downshifting emission around 1540 nm, for which conventional LIR readout is constrained by the small energy separations between Stark sublevels [41,42,43,44,45,46,47,48,49,50,51,52,53,54,55,56,57,58,59,60,61,62,63,64]. This combination allows the full-spectrum approach to be evaluated against both an established ratiometric thermometer and a more challenging spectral regime while retaining the same host and dopant composition.

We use the same temperature-dependent spectral datasets to compare classical Boltzmann-type LIR, second-order LIR2 calibration, and a representative set of full-spectrum regression models. To assess genuine predictive performance, the models are evaluated not only at temperatures included in the calibration grid but also at held-out interpolation temperatures that are never used during training. Finally, we use SHAP-based feature attribution analysis to determine whether the spectral regions that drive the best-performing ML model correspond to physically meaningful Er^3+^ emission features. This combined benchmarking and interpretability framework evaluates predictive performance and examines whether the spectral regions contributing to temperature prediction are consistent with known temperature-sensitive Er^3+^ emission features.

The applicability of full-spectrum thermometry extends beyond YOF:Er^3+^/Yb^3+^. Our previous study demonstrated PCA-based temperature readout, support vector regression, and Gaussian process regression using the emission spectra of Mn^5+^-activated Sr_5_(VO_4_)_3_Cl [29], establishing applicability to a transition-metal emitter. More recent studies demonstrated full-spectrum thermometry in Ce^3+^/Yb^3+^-doped Y_3_Al_5_O_12_ [65] and Yb^3+^-doped YVO_4_ [7], including evaluation at temperatures excluded from training. Together, these results support the applicability of the spectrum-to-temperature approach across different hosts and emitting-ion classes. Its implementation requires a reproducible temperature-dependent spectral response and material-specific calibration and validation; a fitted model is not assumed to transfer directly between materials.

Practical luminescence spectra may contain additive contributions from stray light, scattering and detector offsets [66]. Such contributions can perturb integrated intensity ratios and can also shift predictions from full-spectrum regression models. We therefore compare LIR-based and full-spectrum methods under two controlled perturbations—a linear baseline and a Rayleigh-scattering-shaped background—and examine whether matched augmentation of the training spectra reduces the resulting temperature-prediction bias. Because the same background families are used for training and testing, this experiment evaluates tolerance to represented synthetic backgrounds rather than general resistance to unmodelled measurement interference.

## 2. Materials and Methods

### 2.1. Materials

YOF was selected as a chemically stable, phase-pure, and sufficiently bright low-phonon host for Er^3+^/Yb^3+^ emission, providing a suitable platform for testing the general principle of full-spectrum thermometric readout [67,68]. Based on our previous study, the optimal dopant concentrations of 1 mol% Er^3+^ and 10 mol% Yb^3+^ were selected, and the corresponding YOF sample was synthesized using the modified Pechini sol–gel method described in Ref. [69] (the chemicals used and manufacturers are provided in the ESI). Briefly, stoichiometric amounts of rare earth nitrates (0.25 mmol) were dissolved in water (90 mL), and citric acid and ethylene glycol were added as chelating and complexing agents. Subsequently, an aqueous ammonium fluoride solution (0.31 mmol in 10 mL H_2_O) was added dropwise under continuous stirring at 80 °C to form the precursor gel. The gel was then calcined in air at 700 °C for 4 h to obtain crystalline YOF nanophosphors.

### 2.2. Methods

Luminescence measurements in the visible (VIS) and near-infrared (NIR) regions were conducted using an Ocean FX UV–VIS spectrometer (Orlando, FL, USA) (signal-to-noise ratio: 2000:1) and an Ocean Insight NIRQuest+ spectrometer (Orlando, FL, USA) (signal-to-noise ratio: 12,000:1), respectively, with a 940 nm laser as the excitation source. The spectral datasets used for temperature readout comprised 175 wavelength channels spanning 505.014–579.592 nm in the VIS region and 160 channels spanning 1451.156–1699.260 nm in the NIR region. The wavelength grids were slightly nonuniform, with adjacent-channel spacing of 0.424–0.433 nm for VIS and 1.547–1.574 nm for NIR. The training and test datasets used identical wavelength grids within each spectral region. We recorded temperature-dependent photoluminescent spectra from 300 K to 450 K using a MicrOptik nitrogen-cooled heating-cooling stage (Rijksweg 14a 4511PJ Breskens, The Netherlands) for temperature control, with 0.1 K accuracy and a heating rate of 1 K/s. Once the system reached a stable temperature, an additional 2 min waiting period was applied to eliminate transient temperature fluctuations. Afterward, 100 spectra were acquired consecutively at each stabilized temperature under the same experimental conditions. These repeated acquisitions were used to characterize within-session temperature-readout repeatability.

**Figure 2:**
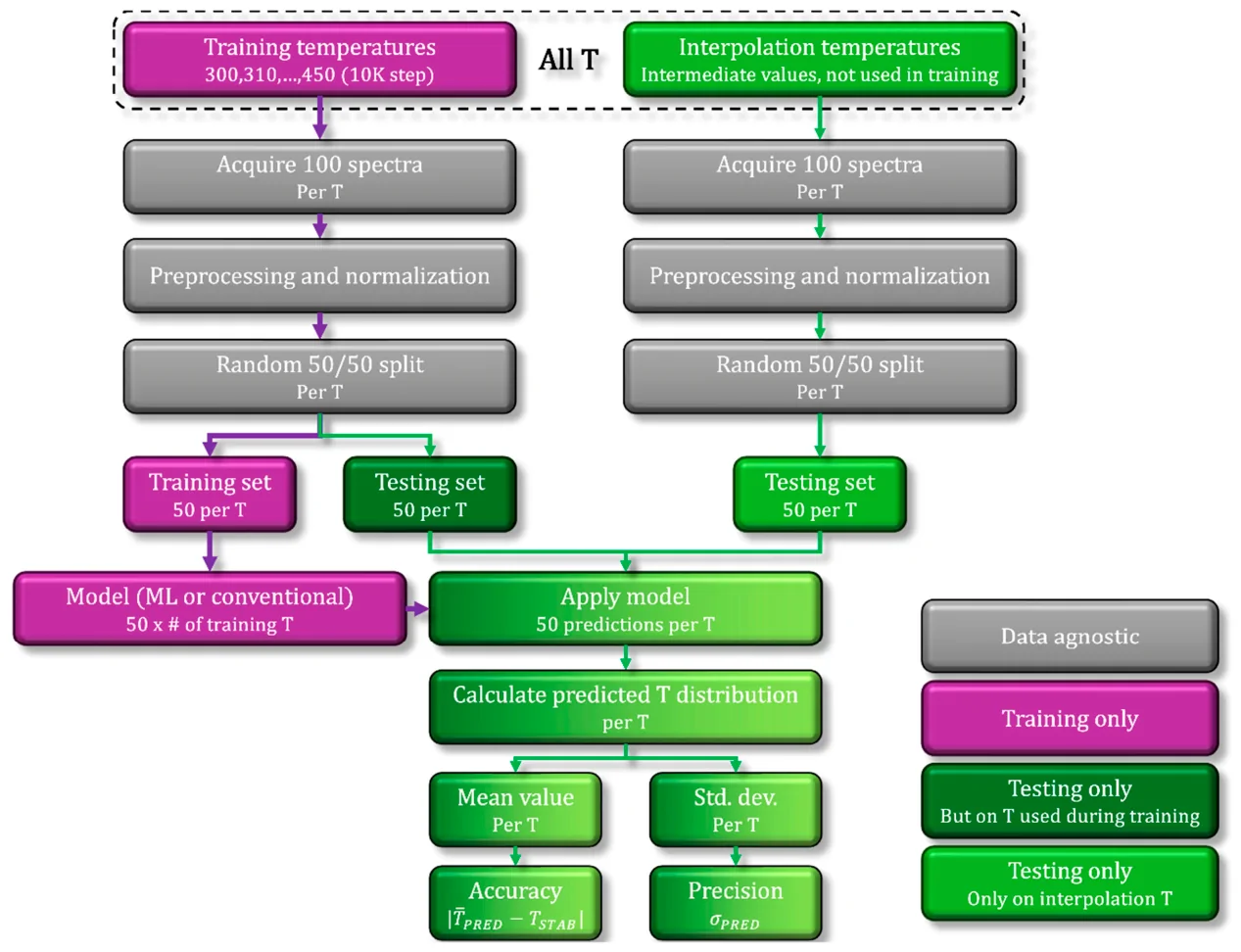
Data-splitting and evaluation protocol for assessing interpolation performance in luminescence thermometry. Emission spectra were acquired at stabilized temperatures across the 300–450 K range. Temperatures sampled at 10 K intervals were used for model training, whereas additional temperatures located between these setpoints were treated as held-out interpolation test temperatures and were never used during training. At each temperature, 100 spectra were recorded and randomly divided into two subsets of 50 spectra. For training temperatures, one subset was used to train the conventional or ML regression model, and the other was retained for testing at temperatures included in the training grid. Spectra acquired at held-out interpolation temperatures were used only for testing. The trained model was applied to each test spectrum to generate 50 predicted temperatures per setpoint, from which the absolute set-point prediction bias, ΔT = |mean(Tpred) − Tstab|, and prediction spread, δT = SD(Tpred), were calculated.

To evaluate the performance of the luminescence thermometry methods, we established a structured data-collection and splitting protocol (see Figure 2).

Luminescent emission spectra were acquired at stabilized temperatures spanning 300–450 K. Temperatures sampled at 10 K intervals were used as training temperatures, whereas additional temperatures located between these intervals were treated as held-out interpolation test temperatures. Spectra acquired at these held-out temperatures were never used during model training and were used exclusively to evaluate the trained model’s ability to predict temperatures at previously unseen setpoints within the calibrated temperature range. To ensure statistically meaningful training and testing, 100 spectra were acquired at each stabilized temperature (this number is implementation-dependent and may be adjusted for different acquisition conditions); 50 were randomly selected to form the training set, and the remaining 50 were reserved for the test set. The ML model was trained exclusively on the spectra collected at training temperatures, using the corresponding 50 training spectra per temperature. Once trained, the model was applied to the test spectra at all stabilized temperatures, yielding, for each temperature, a distribution of 50 predicted values.

At each evaluated setpoint, thermometric performance was quantified by the absolute set-point prediction bias, ΔT = |mean(Tpred) − Tstab|, and the within-setpoint standard deviation of the predicted temperatures, δT = SD(Tpred). These quantities describe agreement of the mean prediction with the reference setpoint and short-term prediction repeatability, respectively. To maintain continuity with terminology commonly used in luminescence thermometry, “accuracy” and “precision” are retained as shorthand for these operational metrics; ΔT and δT do not constitute a complete evaluation of measurement uncertainty. For either metric, a reduction factor is calculated as the reference value divided by the evaluated value, whereas the percentage reduction is calculated as 100 × (1 − evaluated value/reference value). Ratios are calculated from the corresponding mean performance metrics. Comparisons of full-spectrum regression with conventional thermometry use LIR2 as the reference; comparisons assessing training-set augmentation use the same model and normalization trained on the original spectra, evaluated at the same background shape and level. Thermometric performance was evaluated using both test datasets: the test spectra corresponding to the training temperatures and those acquired at the held-out interpolation temperatures. In contrast, thermometric prediction performance was assessed exclusively on the held-out interpolation test set, using 50 spectra per temperature to maintain the same statistical robustness as for the training-temperature evaluation (the remaining 50 spectra acquired at each interpolation temperature were intentionally excluded).

The training and test subsets contained no shared spectra, but they were acquired within the same measurement session. Their separation therefore does not establish independence with respect to session-specific effects. Evaluation at held-out temperatures assesses interpolation within the calibrated range, whereas the within-setpoint prediction spread characterizes short-term repeatability under the investigated conditions. Reproducibility across independent measurement sessions, sample repositioning, and different sample preparations was not evaluated.

### 2.3. Luminescence Intensity Ratio Thermometry

Within the framework of traditional, feature-based temperature read-outs, often referred to as Luminescence Thermometry 1.0, the LIR method stands out as the most extensively researched and practically applied technique [1,9,70].

**Figure 3:**
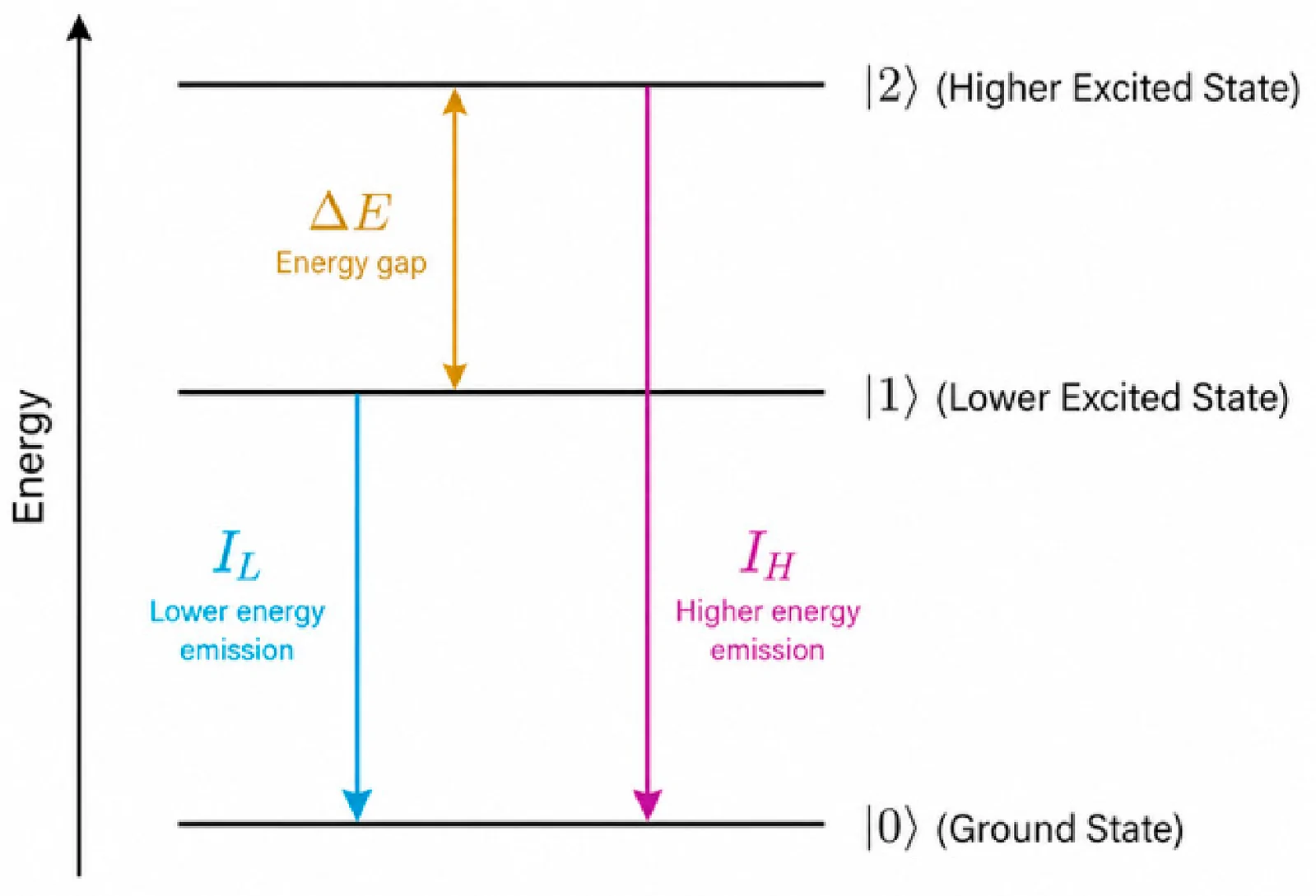
Principle of LIR thermometry based on thermally coupled emitting states. Schematic energy-level representation of a conventional LIR temperature read-out. Two excited states, |1⟩ and |2⟩, are separated by an energy gap (ΔE) and are assumed to be thermally coupled, so that their relative populations follow the Boltzmann distribution. Radiative relaxation from the lower and higher excited states to the ground state, |0⟩, gives two emission bands with integrated intensities I_L_ and I_H_, respectively. The temperature-dependent ratio I_H_/I_L_ forms the basis of conventional ratiometric LT, but it uses only selected spectral features rather than the full emission spectrum.

The widespread use of the LIR method stems from its self-referencing (ratiometric) nature [1,3]. By calculating the ratio between the integrated intensities of two distinct emission bands rather than relying on the absolute intensity of a single band, the LIR approach inherently circumvents severe measurement errors. It is virtually immune to fluctuations in excitation power density, variations in luminescent probe concentration, material inhomogeneities, and optoelectronic drifts in the detection system [1,3]. Furthermore, when compared to time-resolved lifetime measurements, the LIR technique is faster, requires significantly less complex and more cost-effective instrumentation, and is highly adaptable for real-time, two-dimensional thermal imaging [1,3]. The fundamental operating principle of the conventional LIR method relies on the dynamics of thermally coupled energy levels (TCLs) within a luminescent center, typically involving trivalent lanthanide or transition-metal ions [1] (see Figure 3).

When two adjacent excited states are separated by a relatively small energy gap, the available thermal energy is sufficient to promote electrons from the lower to the higher excited state [9]. Provided that the nonradiative transition rates between these two levels significantly exceed their radiative decay rates to the ground state, they establish a rapid thermal equilibrium [71,72]. Because of this equilibrium, the relative population of these emitting states is governed strictly by the Boltzmann distribution [62]. The temperature-dependent LIR can therefore be mathematically modeled by the equation:

(1) $$\mathrm{LIR}(T)=\frac{I_{H}}{I_{L}}=B\cdot e^{-\frac{\Delta E}{k_{B}T}},$$

where I_H_ and I_L_ are the emission intensities corresponding to the thermalized levels of higher and lower energy, B is a temperature-invariant pre-exponential constant that depends on the host material’s properties, *k_B_* is Boltzmann’s constant, and T is the absolute thermodynamic temperature [3,73]. When rewritten, a linear dependence between log(LIR) (log stands for the natural logarithm) and the inverse temperature becomes apparent with a slope proportional to ∆*E* (Equation (2)):

(2) $$\log\left[\mathrm{LIR}(T)\right]=\log\left(B\right)-\frac{\Delta E}{k_{B}}\frac{1}{T}.$$

As the temperature of the material increases, the higher-energy level becomes increasingly populated, causing its emission intensity to grow relative to the lower-energy level. Consequently, the relative sensitivity of this Boltzmann-type thermometer is directly proportional to the energy gap (ΔE/(k_B_T^2^)), meaning the sensor’s fundamental precision is inherently linked to the specific energy level structure of the chosen luminescent dopant [3].

### 2.4. Full-Spectrum Regression Converts Spectral Complexity into Temperature Information

Prior to implementation within the LT2.0 ML framework, the emission spectra were normalized to reduce non-temperature-related intensity variations and improve comparability between spectra. Three normalization procedures were evaluated: maximum normalization, area normalization, and ZSCORE standardization (the standard normal variate transform) (Table 1) [74,75,76].

To evaluate the LT2.0 framework, we implemented PCA-based thermometry alongside a representative set of regression methods. PCA was used for dimensionality reduction, followed by distance-based temperature estimation from the first principal component (PC1). The regression methods comprised partial least squares regression (PLS), random forest regression (RF), least squares boosting (LSB), support vector regression (SVR), Gaussian process regression (GPR), and artificial neural networks (ANN). These algorithms were selected not to optimize a single model architecture, but to assess whether using full-spectrum information improves thermometric performance across different classes of data-driven regression models. Hyperparameters for RF, LSB, SVR, and GPR were selected using MATLAB’s (V. R2026a) Bayesian optimization routines with cross-validated prediction error as the objective. ANN architectures were compared by grid search and selected according to training-set RMSE, without a separate internal validation subset. Hyperparameter selection was restricted to the training data; the reserved test spectra and held-out interpolation temperatures were used only for subsequent performance evaluation. Details of the individual ML algorithms [27,77,78,79,80,81,82,83,84,85,86,87], hyperparameter settings (Table S1), and implementation are provided in the ESI.

For each model, the input consisted of the complete normalized emission spectrum, represented as a vector of wavelength-dependent intensities, and the output was the independently measured stabilized temperature. The models were trained only on spectra acquired at the predefined training temperatures, using the training subset described in Figure 2. After training, the same model was applied without further adjustment to test spectra acquired both at temperatures included in the training grid and at held-out interpolation temperatures that were never used during model training. This protocol allowed the models to be evaluated not only for repeatability at calibrated setpoints, but also for interpolation capability within the calibrated temperature range.

The conventional LIR and modified second-order LIR2 models were evaluated using the same data-splitting and prediction protocol, enabling a direct comparison between feature-based thermometry and full-spectrum regression on identical experimental data. For LIR-based models, temperature was inferred from the calibrated relationship between the selected intensity ratio and temperature. For LT2.0 models, temperature was predicted directly from the complete normalized spectrum. In all cases, the test spectra at each stabilized temperature produced a distribution of predicted temperatures, from which accuracy and precision were calculated as described above.

## 3. Results and Discussion

The YOF: Er^3+^, Yb^3+^ nanophosphor used as the model thermometric probe was confirmed by X-ray diffraction (details are provided in the ESI) to crystallize in the rhombohedral YOF phase, with no detectable secondary phases (Figure S1, ESI). This phase-pure material, therefore, provides a stable platform for comparing conventional feature-based LIR thermometry with full-spectrum ML regression.

**Figure 4:**
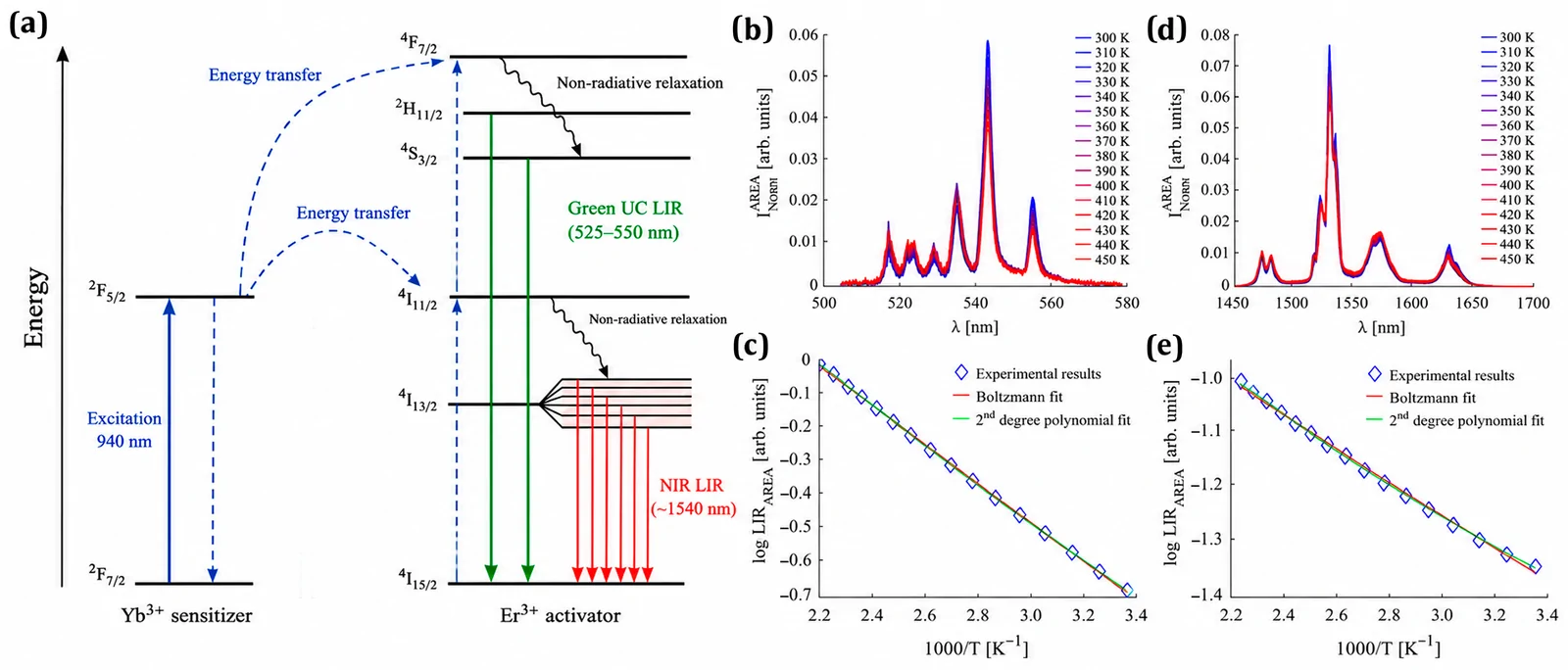
Spectroscopic basis of visible and near-infrared LIR thermometry in YOF: Er^3+^, Yb^3+^ nanoparticles. (**a**) Schematic energy-level diagram showing 940 nm excitation of Yb^3+^ sensitizer ions, energy transfer to Er^3+^ activator ions, non-radiative relaxation pathways, and the radiative transitions used for ratiometric temperature read-out. Visible green UC emission from the thermally coupled Er^3+ 2^H_11/2_ and ^4^S_3/2_ levels to the ^4^I_15/2_ ground state provides the LIR signal in the 525–550 nm range, whereas downshifting emission from the ^4^I_13/2_ manifold to ^4^I_15/2_ provides the NIR LIR signal around 1540 nm. (**b**) Area-normalized green UC emission spectra recorded between 300 and 450 K under 940 nm excitation. (**c**) Temperature dependence of the VIS-region LIR, plotted as log(LIR_AREA_) versus 1000/T, together with Boltzmann and second-degree polynomial fits. (**d**) Area-normalized NIR Er^3+^ emission spectra recorded over the same temperature range. (**e**) Temperature dependence of the NIR LIR, fitted using the same Boltzmann and second-degree polynomial models. The comparison establishes conventional feature-based LIR thermometry in both spectral regions as the benchmark against which full-spectrum ML regression is evaluated.

In the VIS region, the emission spectra recorded at different temperatures under 940 nm excitation show the characteristic Er^3+^ green UC emissions originating from the excited ^2^H_11/2_ and ^4^S_3/2_ levels to the ^4^I_15/2_ ground level. This is enabled by energy transfer from the ^2^F_5/2_ level of Yb^3+^ to Er^3+^ (see energy diagram in Figure 4a) [88,89]. An increase in the relative intensity of the ^2^H_11/2_ emission with increasing temperature is observed and can be attributed to thermal coupling between the ^2^H_11/2_ and ^4^S_3/2_ excited states (Figure 4b). Normalized emissions were used to fit the experimental data with both the conventional Boltzmann-type equation (LIR) and the modified second-order polynomial equation (LIR2), as shown in Figure 4c. The temperature dependence of the intensity ratio between the two green emissions shows nearly ideal Boltzmann-type behavior, consistent with numerous reports in the literature. To achieve the best possible performance within the LT1.0 framework, the dependence was also fitted with a second-order polynomial function to account for slight deviations from linearity arising from weak overlapping temperature-dependent emissions from higher-energy excited states:

(3) $$\log\left[\mathrm{LIR}2(T)\right]=\log\left(B\right)-\frac{\Delta E}{k_{B}}\frac{1}{T}+\mathrm{constant}\cdot \frac{1}{T^{2}}.$$

We rigorously compare the classical Boltzmann-type LIR (Equation (2)) and a purely mathematical regression model based on the second-order polynomial with respect to 1/*T* (LIR2) (Equation (3)) with a range of ML algorithms. The modified LIR2 model was used to account for small deviations from the purely linear Boltzmann behavior of the log(LIR(1/T)) curve, primarily due to peaks of interest overlapping with other spectral lines at lower temperatures, where thermalization is weakest.

In the NIR region, upon excitation at 940 nm, a single downshifting emission corresponding to the ^4^I_13/2_ → ^4^I_15/2_ transition of Er^3+^ is observed, centered at approximately 1540 nm (Figure 4d) [90,91]. The broad ^4^I_13/2_ → ^4^I_15/2_ emission band consists of multiple overlapping components that can be divided into two groups. The peaks located between 1450 and 1500 nm and above 1550 nm increase in intensity with temperature, as they originate from the higher-energy Stark sublevels of the ^4^I_13/2_ level, whereas the peaks between 1500 and 1550 nm arise from the lower-energy Stark sublevels [59]. Analogously to the VIS region approach for the Er^3+^/Yb^3+^ system, the ratio of these intensities is used for the Boltzmann-type LIR and a modified second-order polynomial LIR (LIR2) (Figure 4e).

A broad range of ML techniques was employed within the LT2.0 framework. Because the full-spectrum approach is nonparametric, the same evaluation was applied to the conventional LIR and LIR2 feature-based approaches to compare their outputs with those of the full-spectrum methods. As a result, a distribution of predicted temperatures was obtained at each stabilized temperature, regardless of whether data at that temperature were used in training or whether it was a held-out interpolation temperature. These distributions were characterized by two key figures of merit: accuracy (ΔT) and precision (δT), as previously defined. Figures S2 and S3 (ESI) show set-point-wise results for the quadratic LIR calibration after MAX normalization and for the GPR model selected for SHAP analysis after ZSCORE normalization. Table S2 (ESI) summarizes ΔT and δT averaged over all evaluated setpoints, combining in-grid and held-out interpolation tests. Because these averages do not isolate interpolation performance, Table 2 reports the corresponding quantities calculated only at held-out interpolation temperatures. Once the predictive model is trained and validated, determining temperature requires only a single emission spectrum.

The left table summarizes results obtained using green VIS UC emissions, whereas the right table presents results for downshifting NIR emissions. These results provide a descriptive comparison under the investigated conditions; small numerical differences between methods should not be interpreted as statistically established performance differences. In both cases, the ΔT and δT achieved with the conventional LIR approach are comparable to values commonly reported in the literature [41,42,43,44,45,46,47,48,49,50,51,52,53,54,55,56,57,58,59,60,61,62,63,64]. Second-order polynomial calibration (LIR2) reduced the mean absolute set-point bias relative to the Boltzmann-type LIR calibration in both spectral regions, while producing only small changes in the within-setpoint prediction spread. LIR2 therefore provides the best-performing conventional LIR benchmark evaluated here, rather than an established limit of ratiometric thermometry. Relative to LIR2, the largest reductions in absolute bias and prediction spread across the evaluated full-spectrum models are approximately 2.5-fold and 24%, respectively, in the VIS region, and 2.2-fold and 8.6-fold in the NIR region. These comparisons use the lowest value obtained for each metric across the evaluated models and normalization procedures; the best bias and spread need not be achieved by the same model.

Having established the predictive advantage of full-spectrum regression, we next investigated whether the spectral information exploited by ML models corresponds to physically meaningful Er^3+^ emission features. To address this, we employed explainable artificial intelligence (XAI), which transforms an accurate yet difficult-to-interpret ML model into a spectroscopically interpretable framework. XAI identifies spectral regions contributing to temperature prediction, allowing their correspondence with temperature-sensitive Er^3+^ emission features to be examined. This provides a spectroscopic interpretation of the model’s predictions but does not independently establish physical causality or exclude spurious statistical correlations.

For demonstration purposes, the XAI framework was implemented using the GPR model, which was selected because it ranked among the most accurate regression models, achieving the highest performance in the VIS and near-best performance in the NIR spectral range. Although the GPR model has a transparent probabilistic formulation with interpretable kernel hyperparameters and analytical uncertainty estimates [83], its predictions arise from nonlinear kernel interactions across the entire spectrum rather than explicit contributions of individual wavelengths. Consequently, post hoc XAI interpretation remains essential for identifying the spectral origin of the model predictions.

For this purpose, SHapley Additive exPlanations (SHAP) were applied directly to the trained GPR model without modifying its Gaussian-process formulation. SHAP was selected because it is model-agnostic and provides wavelength-resolved feature attributions independent of model architecture [92]. Unlike linear regression coefficients or PCA/PLS loadings, SHAP explains the predictions of the final trained model, thereby capturing nonlinear and nonlocal spectral-temperature relationships encoded by full-spectrum regression. Furthermore, SHAP provides both local explanations for individual spectra and global feature-importance (GFI) distributions obtained by averaging absolute SHAP values across the test set. This enables temperature-dependent feature-importance maps and global SHAP spectra, allowing ML predictions to be interpreted directly in spectroscopic terms.

**Figure 5:**
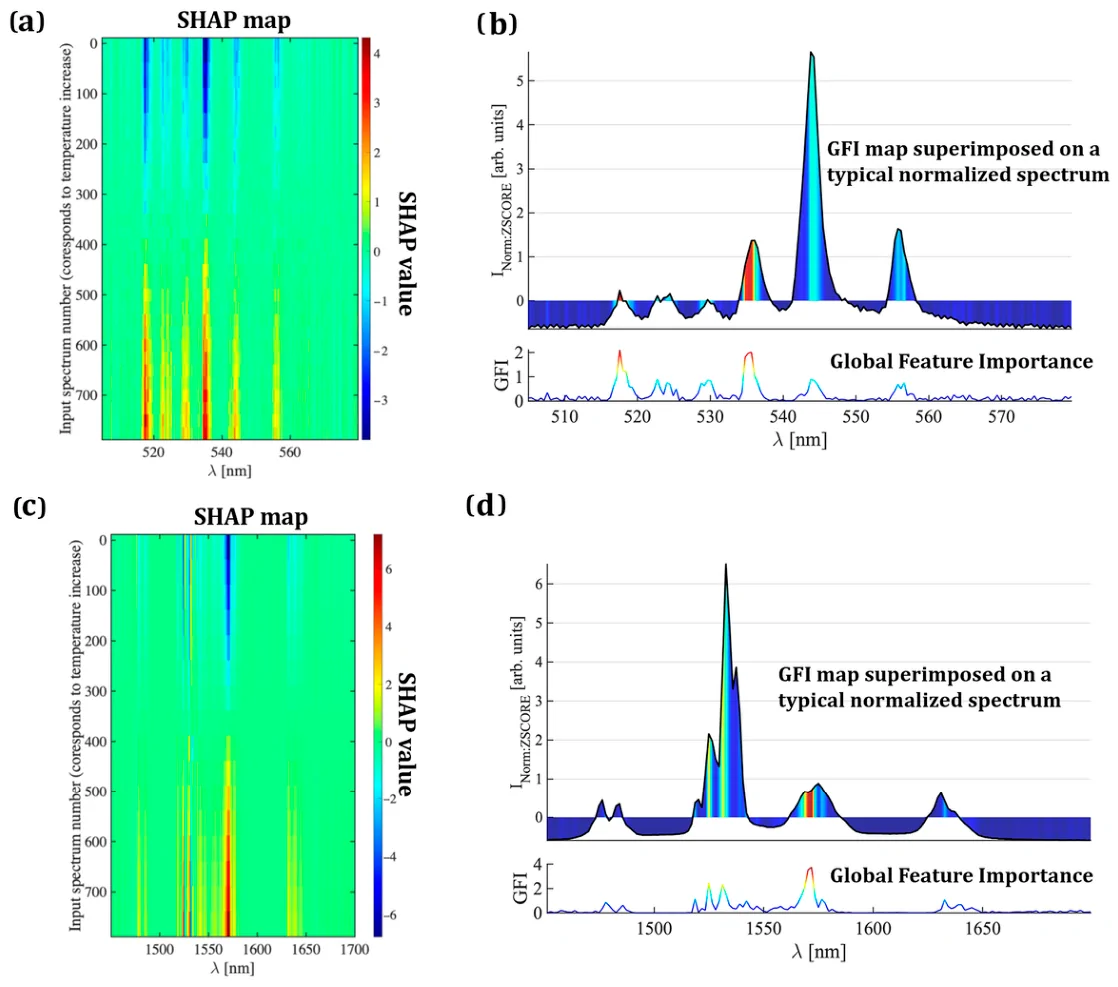
Example of XAI analysis of the GPR model with ZSCORE normalization. (**a**,**c**) Temperature-dependent SHAP maps for the green VIS UC and NIR spectra, respectively (higher spectral numbers correspond to higher temperatures). (**b**,**d**) GFI (bottom), represented by the average absolute SHAP value at each wavelength, together with the corresponding average ZSCORE-normalized emission spectra (top).

Originating from cooperative game theory, SHAP quantifies the marginal contribution of each wavelength to the predicted temperature. SHAP values were calculated for all spectra, producing attribution maps (Figure 5a,c) that reveal how spectral importance evolves with temperature. Averaging the absolute SHAP values at each wavelength yielded the GFI spectrum (Figure 5b,d).

Because adjacent wavelength channels are highly correlated, SHAP values should be interpreted as attribution maps highlighting spectrally informative regions rather than independent causal contributions of individual wavelengths.

The SHAP analysis of the GPR model with ZSCORE normalization on the VIS dataset (Figure 5b) assigns the greatest feature importance to emission regions below 540 nm, consistent with the temperature-dependent response of the thermally coupled Er^3+^ excited states. The attribution distribution also shows an asymmetry across the emission bands: the higher-energy sides of the high-energy peaks contribute more strongly to temperature prediction, whereas the opposite trend is observed for the lower-energy transitions. These patterns indicate how the fitted model uses spectral information and are consistent with a spectroscopic basis for its predictions; they do not independently demonstrate the physical mechanisms responsible for these features.

For conventional Er^3+^ thermometry based on the thermally coupled ^2^H_11/2_ and ^4^S_3/2_ levels, the Boltzmann relative sensitivity is governed by their energy separation, which varies comparatively little among hosts. Host substitution therefore offers limited scope for increasing this intrinsic sensitivity, although improvements in emission brightness, thermal-quenching behavior, and stability can improve measurement repeatability and extend the usable temperature range. Examples include Yb^3+^/Er^3+^-based glass ceramics and core–shell structures that exploit contrasting thermal-expansion behavior to modify temperature-dependent upconversion emission [93,94]. LT2.0 provides a complementary route by extracting temperature information distributed across the emission spectrum. The present results demonstrate that substantial improvements in temperature prediction can be achieved through spectral readout without changing the thermometric material.

### Robustness of Ratiometric and Machine-Learning Thermometry to Spectral Background

ML models are expected to be more resilient to background interference than LIR, given their access to the full spectrum; it is less clear whether this potential translates into practice. In particular, it remains unclear whether simply introducing background into training spectra is sufficient to meaningfully increase a model’s robustness, and how accurate the resulting predictions are in absolute terms. This section addresses these questions through a systematic evaluation of LIR and ML-based approaches under controlled background interference.

**Figure 6:**
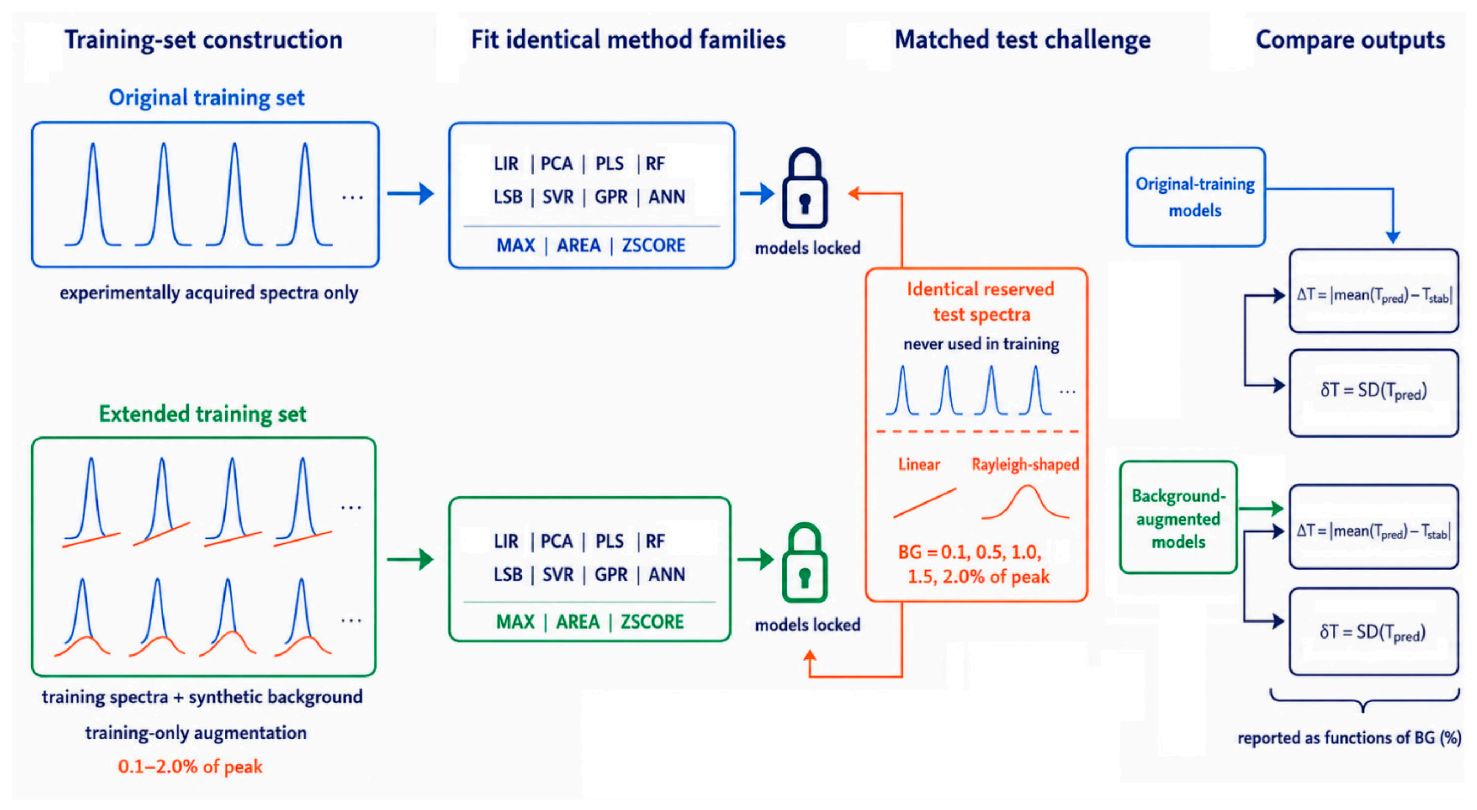
Training-set augmentation and matched synthetic-background evaluation. For each readout method (LIR, PCA, PLS, RF, LSB, SVR, GPR, and ANN) and normalization (MAX, AREA, ZSCORE), two versions were fitted: one using only experimentally acquired training spectra and another using an extended training set generated by adding simulated linear- or Rayleigh-scattering-shaped BG to the training spectra. After fitting, both model versions were locked and applied to the same independently reserved test spectra, to which the corresponding synthetic BG was added at 0.1–2.0% of the peak signal. At each BG level, performance was quantified as absolute bias (accuracy), ΔT = |mean(T_pred_) − T_stab_|, and prediction spread (precision), δT = SD(T_pred_). Because the training and test perturbations were drawn from the same BG families, this protocol evaluates tolerance to matched synthetic BG rather than general resistance to unmodeled experimental interference.

Here, we present in detail the results obtained on VIS spectra. A variable background (BG) was added to experimentally acquired test spectra (0.1%, 0.5%, 1%, 1.5%, and 2% of the peak signal) in two shapes representative of common experimental sources: a linear and a Rayleigh-scattering-shaped BG. As illustrated in Figure 6, two versions of every model were trained: one using only the original, experimentally acquired spectra (the original training set), and one using a training set extended with the same kind of variable BG (the extended training set). The background profiles, amplitude scaling, augmented training-set size, and order of preprocessing are specified in the ESI, Section “Synthetic-background generation and training-set augmentation.”

Both versions were evaluated on identical test spectra, so any difference between them reflects only what each model was allowed to learn from. This procedure was applied using all three normalization methods (MAX, AREA, and ZSCORE). Absolute bias (ΔT) and prediction spread (δT) are reported as functions of the variable BG.

**Figure 7:**
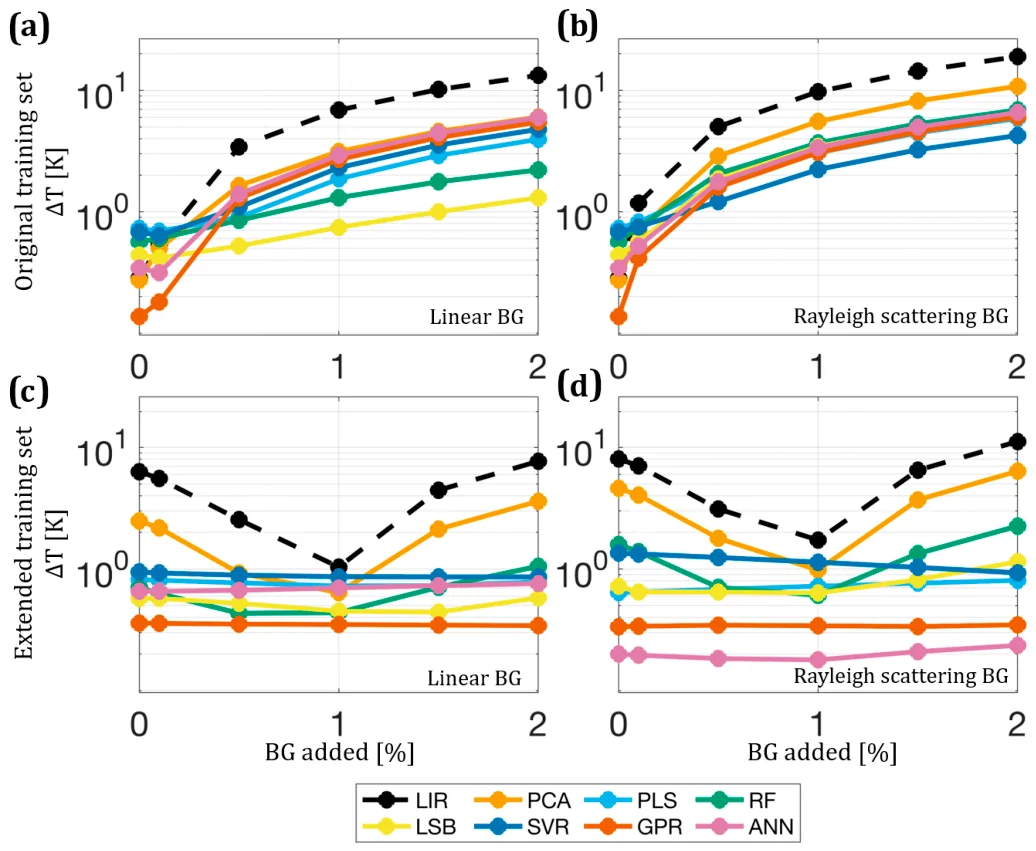
Absolute bias, ΔT (accuracy) for all eight methods. Trained on the original spectra: (**a**) linear, (**b**) Rayleigh-scattering BG, and on the extended training set: (**c**) linear, (**d**) Rayleigh-scattering BG.

With the original training set, every method’s accuracy worsens as BG is added to the testing set, though by varying degrees (Figure 7a,b; MAX normalization is shown, while AREA and ZSCORE normalizations are presented in Figures S4a,b and S5a,b in the ESI). LIR is most severely affected, with its error rising from 0.28 K with no BG to 13.3 K and 19.0 K with a linear and Rayleigh scattering BG at the highest level tested, respectively. The seven ML methods degrade as well, but far less; GPR, for instance, stays under 6 K even in the worst case, and several methods remain below 2–3 K throughout.

This changes substantially once the extended training set is included (Figure 7c,d, Figure S4c,d and Figure S5c,d in the ESI). Most ML methods now remain well below 1 K across the entire BG range: GPR’s error, for example, is confined to 0.32–0.36 K regardless of how much BG is present, and SVR, PLS, and ANN show similarly small, nearly flat errors. LIR performance also improves, but far less; its error still reaches 7.7–11.2 K at the highest BG level (2%), only a modest reduction from its original performance. For all methods, this improvement in robustness comes at the cost of degraded baseline absolute bias (in the absence of any BG). In absolute terms, this degradation is highest for LIR (from 0.28 K to 6.3–8.1 K) compared to ML methods, with some still maintaining sufficiently good absolute bias (below 1 K).

**Figure 8:**
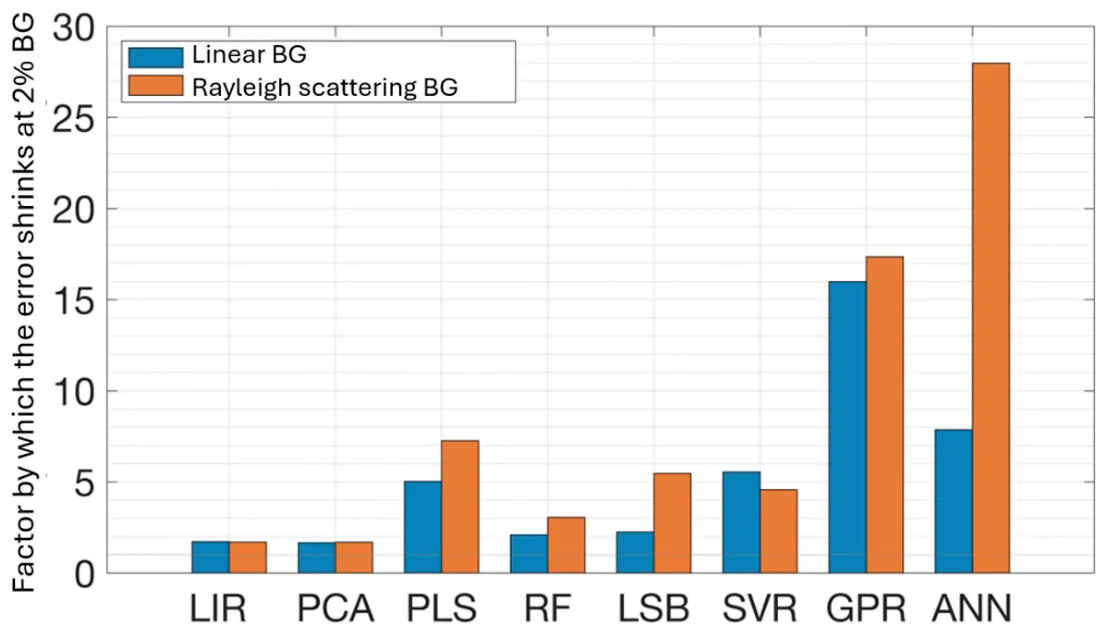
Absolute-bias reduction factor, ΔT_original_/ΔT_extended_, at 2% background (BG), using MAX normalization. LIR and PCA improve by about a factor of 1.7; the remaining ML methods improve considerably more, typically by a factor of 5 to 20.

The effect of training-set augmentation was quantified as the ratio of absolute bias obtained with the original training set to that obtained with the extended training set, evaluated at 2% BG for the same method, normalization, and background shape (Figure 8 and Figure S6, ESI). LIR and PCA improve performance only modestly, by about a factor of 1.7 regardless of BG type. The remaining ML methods improve performance considerably more, typically by a factor of 5 to 20.

**Figure 9:**
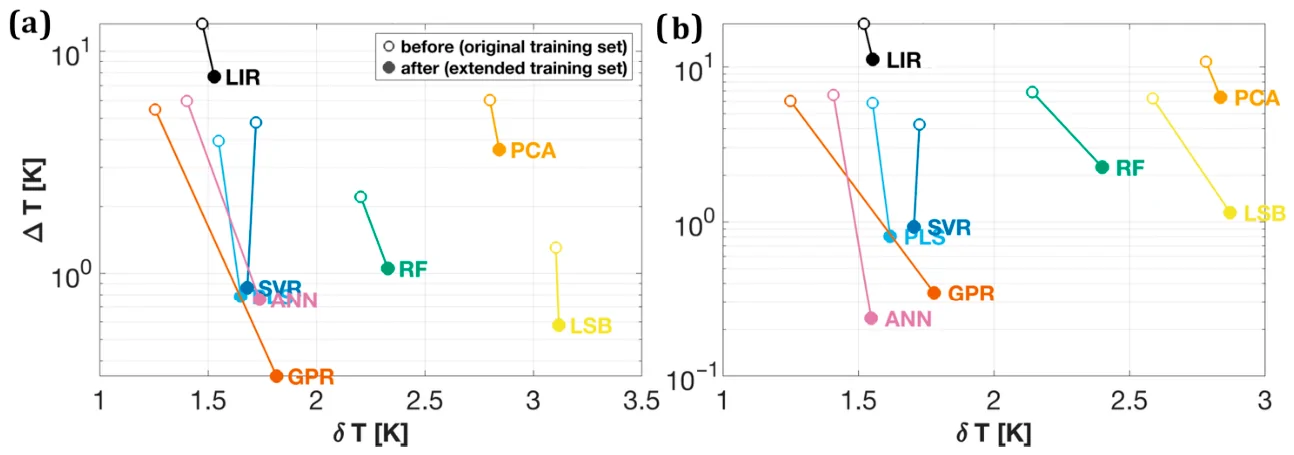
Absolute bias, ΔT (accuracy; vertical axis, log scale) and prediction spread, δT (precision; horizontal axis), at 2% of: (**a**) linear and (**b**) Rayleigh-scattering BG (MAX normalization, lower values are better).

The prediction spread, δT (precision), behaves differently from accuracy throughout this analysis. Across all methods and both training sets (original and extended), δT changes only modestly with BG level (Figures S7–S9, ESI). The BG mainly introduces a systematic offset in the predicted temperature rather than additional scatter around it. Because of this, the more informative comparison is not how δT changes with background, but how it compares across methods once training is extended, alongside the corresponding accuracy gain. Figure 9a,b plot each method’s accuracy and precision at 2% for linear and Rayleigh-scattering BG, respectively (MAX normalization; AREA and ZSCORE are given in Figures S10 and S11 of the ESI). Each method is represented with two dots connected by a line. An open circle represents the original training set, while a filled circle represents the extended training set values. Most ML methods move straight down, with a large reduction in ΔT and little to no change in δT. GPR is the one exception worth noting individually: its prediction spread, δT, increases from approximately 1.25 to 1.8 K, corresponding to a 44% increase, its absolute bias, ΔT, is reduced by a factor greater than 15 compared to the original training model. Even after this shift, GPR’s δT remains better than PCA’s, RF’s, or LSB’s and comparable to the rest, while maintaining much better ΔT, placing it among the strongest performers overall.

**Figure 10:**
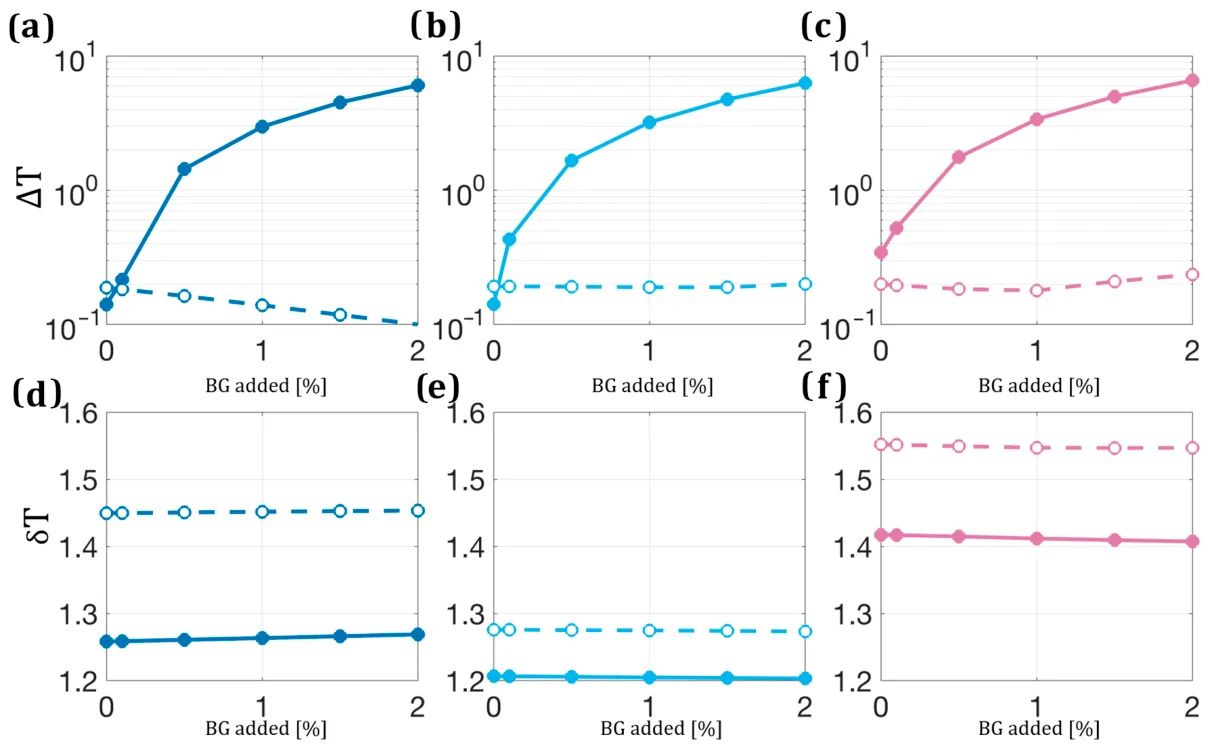
The three largest improvements of absolute bias (accuracy) found, shown across the full BG range. Solid circles/lines: original training set; open circles/dashed lines: extended training set. (**a**) ΔT and (**d**) δT for SVR (ZSCORE normalization, linear BG), (**b**) ΔT and (**e**) δT for PLS (ZSCORE normalization, Rayleigh-scattering BG), and (**c**) ΔT and (**f**) δT for ANN (MAX normalization, Rayleigh-scattering BG). In all three cases, extending the training set makes accuracy nearly independent of BG, with no visible cost to precision.

The three cases showing the largest reductions in absolute bias (Figure 8 and Figure S6, ESI) are presented in Figure 10a–c for SVR, PLS, and ANN, respectively. In each case, extending the training set converts a method whose ΔT clearly degrades with the introduction of background into one that performs consistently across the entire tested range, with δT virtually unaffected (Figure 10d–f), underscoring the effectiveness of background-aware training as a robust and low-cost strategy for improving real-world thermometric performance.

The same pattern holds in the NIR dataset (Figures S12–S14, ESI): every method’s ΔT degrades with increasing background when trained only on experimentally acquired spectra, with LIR degrading far more severely than any ML method. Extending the training set delivers a large, consistent ΔT reduction for GPR, SVR, ANN, and PLS, while LIR and PCA performance improve only marginally and at a real cost to their baseline performance. Prediction spread, δT, remains largely insensitive to background level throughout.

Taken together, the VIS and NIR results tell the same story: LIR cannot be trained into robustness to background, but several ML methods already have the flexibility to achieve it. LIR’s comparatively small performance improvement from an extended training set reflects a structural limit rather than a shortcoming of the training procedure itself. LIR always computes temperature from the same fixed ratio between two spectral bands, so retraining shifts where that ratio maps onto the temperature scale, but it cannot identify and discount the background part of the spectrum, since the method never looks at the rest of the spectrum. ML regressors, by contrast, use the full spectral shape and so have the flexibility to learn which features are diagnostic of temperature, and which are not. This is why several of them, particularly SVR, PLS, and ANN under the right normalization, can nearly eliminate the effect of background on ΔT, without a meaningful cost to δT.

## 4. Conclusions

We have evaluated Luminescence Thermometry 2.0 as a full-spectrum temperature read-out framework that replaces manual selection of isolated spectral features with direct regression from the complete emission spectrum. Using Er^3+^/Yb^3+^ co-doped YOF nanoparticles as a model system, we benchmarked this approach against conventional Boltzmann-type LIR and second-order regression LIR2 thermometry using identical visible upconversion and near-infrared downshifting spectral datasets. The full-spectrum models substantially improved thermometric prediction at held-out interpolation temperatures, showing that feature-based analysis discards temperature information that can be recovered and used to improve both accuracy and precision.

The improvement was especially pronounced in the near-infrared spectral region, where conventional LIR thermometry is constrained by the small energy separation between Er^3+^ Stark sublevels. In this regime, full-spectrum regression exploited distributed spectral changes across the emission band rather than relying on a single manually defined intensity ratio. SHAP-based feature-attribution analysis identified emission regions contributing to the GPR predictions that are consistent with known temperature-sensitive Er^3+^ spectroscopic features.

Controlled tests with synthetic linear and Rayleigh-scattering-shaped backgrounds showed that full-spectrum models trained only on unmodified spectra can develop substantial background-induced set-point bias. Matched augmentation of the training spectra reduced this bias for several regression methods, although the effect depended on the model and normalization and was sometimes accompanied by increased zero-background bias or within-setpoint spread. These results demonstrate tolerance to background families represented during training; they do not establish general resistance to detector noise, instrumental drift, or unmodelled spectral interference.

These results establish full-spectrum regression as a general strategy for improving luminescence thermometry without requiring the development of new probe compositions. More broadly, they suggest that complex spectral changes, including peak overlap and nonlinear temperature-dependent distortions, can be treated as useful thermometric information when analyzed with appropriate data-driven models. This framework should be transferable to other luminescent probes, spectral ranges, and sensing architectures, providing a route toward more accurate, precise, and interpretable optical temperature read-out.

## Figures and Tables

**Table 1:** Spectral normalization strategies used before full-spectrum machine-learning regression. Summary of the preprocessing normalization approaches applied to the emission spectra before implementing the Luminescence Thermometry 2.0 framework. These normalization procedures reduce non-temperature-related intensity variations and improve the comparability of spectra prior to regression analysis.

**Maximum normalization**	intensity-scaling normalization method in which all spectral values are normalized to the maximum signal intensity value
**Area normalization**	integral-based normalization method, where each value is scaled relative to the total spectral area or summed intensity values
**ZSCORE normalization**	statistical standardization method that centers spectral data such that it has a 0 mean value and a standard deviation of 1

**Table 2:** Thermometric prediction performance at held-out interpolation temperatures. Accuracy, ΔT, and precision, δT, obtained from VIS UC and NIR downshifting emission spectra using conventional LIR-based thermometry and full-spectrum ML regression. At each held-out interpolation temperature, ΔT was calculated as the absolute difference between the mean of 50 temperature predictions and the stabilized reference temperature, whereas δT was calculated as the standard deviation of those predictions. The tabulated values are the arithmetic means of these setpoint-specific quantities over the held-out interpolation temperatures, which were excluded from model training. Here, δT describes within-setpoint prediction spread; confidence intervals for the tabulated means are not reported. Results are shown for maximum, area, and ZSCORE spectral normalization. Entries marked with **^†^** represent the best conventional LIR-family performance, and the best ML performance within each spectral range is marked in bold.

VIS Spectral Range, Prediction Performance Absolute Bias, ΔT [K] (Accuracy) and Prediction Spread, δT [K] (Precision)							NIR Spectral Range, Prediction Performance Absolute Bias, ΔT [K] (Accuracy) and Prediction Spread, δT [K] (Precision)						
Norm.→ Method	MAX		AREA		ZSCORE		Norm.→ Method	MAX		AREA		ZSCORE	
	ΔT	δT	ΔT	δT	ΔT	δT		ΔT	δT	ΔT	δT	ΔT	δT
**LIR**	0.93	1.63	0.93	1.63	—	—	**LIR**	1.43	1.25	1.43	1.25	—	—
**LIR2**	0.28 **^†^**	1.60 **^†^**	0.28 **^†^**	1.60 **^†^**	—	—	**LIR2**	0.26 **^†^**	1.21 **^†^**	0.26 **^†^**	1.21 **^†^**	—	—
**PCA**	0.27	2.91	0.61	3.09	0.21	1.27	**PCA**	0.59	0.34	0.36	0.67	0.43	**0.14**
**PLS**	0.73	1.56	1.13	1.98	0.14	**1.21 **	**PLS**	0.23	0.28	0.36	0.66	0.26	0.15
**RF**	0.56	2.28	0.92	2.75	0.73	1.94	**RF**	1.37	0.83	1.25	0.53	1.58	0.29
**LSB**	0.44	3.34	0.80	4.29	0.55	3.77	**LSB**	2.02	1.89	2.30	1.80	2.03	1.68
**SVR**	0.68	1.74	0.91	2.40	0.14	1.26	**SVR**	0.17	0.16	0.15	0.20	0.22	**0.14**
**GPR**	0.14	1.26	0.17	1.24	**0.11**	1.24	**GPR**	0.17	0.15	0.14	**0.14**	0.17	0.15
**ANN**	0.34	1.42	0.56	1.39	0.41	1.55	**ANN**	0.17	0.16	0.16	0.16	**0.12**	0.16

* Results shown for held-out interpolation temperatures. Lower ΔT and δT values indicate better performance.

## Data Availability

The dataset supporting the conclusions of this article is available in the Zenodo repository: https://zenodo.org/records/21527081 (accessed on 28 September 2026).

## Supplementary Materials

The following supporting information can be downloaded at: https://www.mdpi.com/article/10.3390/nano16191238/s1, Figure S1: X-ray diffraction pattern of the YOF: Er^3+^/Yb^3+^ nanophosphor, presented along with the ICDD card No. 00-025-1012 (crystallite size = 154 (7) Å, microstrain = 0.320 (7), and a = b = 3.797 Å, and c = 18.838 (5) Å parameters); Figure S2: VIS spectral range. Predicted vs. stabilized temperatures for a. LIR2 and b. GPR, and calculated accuracy (diamonds) and precision (circles) for c. LIR2 and d. GPR; Figure S3: NIR spectral range. Predicted vs. stabilized temperatures for a. LIR2 and b. GPR, and calculated accuracy (diamonds) and precision (circles) for c. LIR2 and d. GPR; Figure S4: VIS spectral range, AREA normalization. Absolute bias, ΔT (accuracy), for all eight methods, trained on the original spectra: a. linear, b. Rayleigh-scattering background (BG), and on the extended training set: c. linear, d. Rayleigh-scattering background (BG); Figure S5: VIS spectral range, ZSCORE normalization. Absolute bias, ΔT (accuracy) for all eight methods, trained on the original spectra: a. linear, b. Rayleigh-scattering background (BG), and on the extended training set: c. linear, d. Rayleigh-scattering background (BG); Figure S6: Relative improvement in the absolute bias, ΔT (accuracy) at the highest background level (2%) on the extended training set: a. AREA normalization; b. ZSCORE normalization; Figure S7: MAX normalization. Prediction spread, δT (precision) for all eight methods, trained on the original spectra: a. linear, b. Rayleigh-scattering background (BG), and on the extended training set: c. linear, d. Rayleigh-scattering background (BG); Figure S8: AREA normalization. Prediction spread, δT (precision) for all eight methods, trained on the original spectra: a. linear, b. Rayleigh-scattering background (BG), and on the extended training set: c. linear, d. Rayleigh-scattering background (BG); Figure S9: ZSCORE normalization. Prediction spread, δT (precision) for all eight methods, trained on the original spectra: a. linear, b. Rayleigh-scattering background (BG), and on the extended training set: c. linear, d. Rayleigh-scattering background (BG); Figure S10: Accuracy (ΔT, vertical axis, log scale) and prediction spread, δT (precision; horizontal axis), at 2%. a. linear, and b. Rayleigh-scattering background (AREA normalization, lower is better); Figure S11: Absolute bias, ΔT (accuracy; vertical axis, log scale) and prediction spread, δT (precision; horizontal axis), at 2%. a. linear, and b. Rayleigh-scattering background (ZSCORE normalization, lower is better); Figure S12: NIR spectral range, MAX normalization. Absolute bias, ΔT (accuracy) for all eight methods, trained on the original spectra: a. linear, b. Rayleigh-scattering background (BG), and on the extended training set: c. linear, d. Rayleigh-scattering background (BG); Figure S13: NIR spectral range, AREA normalization. Absolute bias, ΔT (accuracy) for all eight methods, trained on the original spectra: a. linear, b. Rayleigh-scattering background (BG), and on the extended training set: c. linear, d. Rayleigh-scattering background (BG); Figure S14: NIR spectral range, ZSCORE normalization. Absolute bias, ΔT (accuracy) for all eight methods, trained on the original spectra: a. linear, b. Rayleigh-scattering background (BG), and on the extended training set: c. linear, d. Rayleigh-scattering background (BG); Table S1: Fitting and hyperparameter settings for the ML methods used; Table S2: Thermometric performance at each stabilized temperature. Accuracy, ΔT, and precision, δT, obtained from VIS UC and NIR downshifting emission spectra using conventional LIR-based thermometry and full-spectrum ML regression. Results are shown for maximum, area, and ZSCORE spectral normalization. Lower ΔT and δT values indicate better accuracy and precision, respectively. References [27,77,78,79,80,81,82,83,84,85,86,87] are also cited in the Supplementary Materials.
