# Supplementary material for: Luminescence Thermometry 2.0: Machine-Learning Temperature Read-Out with Luminescence of YOF: Yb3+, Er3+ Nanoparticles
Source: Nanomaterials (Basel). 2026 Sep 30;16(19):1238. doi: 10.3390/nano16191238 (PMC13667202; doi:10.3390/nano16191238)
Supplement: Supplementary file 1 [file nanomaterials-16-01238-s001.zip › nanomaterials-4593905-supplementary.pdf]

Luminescence Thermometry 2.0: Machine-Learning Temperature Read-Out with Luminescence of YOF: Yb^{3+}, Er^{3+} Nanoparticles

Zoran Ristić [1], Jovana Periša [1], Aleksandar Ćirić [1], Milica Maričić [1], Anđela Rajčić [1], Tamara Gavrilović [1], Željka Antić [1] and Miroslav D. Dramićanin [1,2,*]

[1] Centre of Excellence for Photoconversion, Vinča Institute of Nuclear Sciences—National Institute of the Republic of Serbia, University of Belgrade, P.O. Box 522, 11001 Belgrade, Serbia

[2] National Institute of Research and Development for Electrochemistry and Condensed Matter, INCEMC, Str. Dr. A. Păunescu Podeanu Nr.144, Timisoara 300569, Romania

* Correspondence: dramican@vinca.rs

Synthesis:

For the materials synthesis, the following chemicals were used as received: metal nitrate salts (yttrium(III) nitrate hexahydrate, $Y(NO_3)_3{\times}6H_2O$; erbium (III) nitrate hexahydrate, $Er(NO_3)_3{\times}6H_2O$; ytterbium (III)-nitrate pentahydrate, $Yb(NO_3)_3{\times}5H_2O$; Alfa Aesar, Ward Hill, MA, USA, purity 99.9%, 99.99+, 99.9%, respectively); citric acid - CA ($HOC(COOH)(CH_2COOH)_2$, Sigma Aldrich, St. Louis, MO, USA, ACS reagent, ≥99.5%), ethylene glycol - EG ($HOCH_2CH_2OH$, Sigma Aldrich, St. Louis, MO, USA, anhydrous, 99.8%), and ammonium fluoride- NH_4F (Alfa Aesar, Ward Hill, MA, USA).

Structural characterization by X-Ray Diffraction (XRD):

The powder's crystal structure was analyzed at room temperature using an Automated Multipurpose X-ray Diffractometer (XRD) with Guidance Software (Rigaku SmartLab, Japan). The measurement covered the 20°–90° range with a 0.02° step size and a counting time of 10°/min. Key results of the structural analysis were obtained using PDXL2-Integrated X-ray Powder Diffraction Software (v2.1).

The X-ray diffraction pattern of the YOF: Er^{3+}/Yb^{3+} nanophosphor, along with the fitting parameters, including the crystallite size, is presented in Figure S1. The sample exhibits a

rhombohedral crystal structure (space group *R-3m*, reference card No. ICDD 00-025-1012), with no detectable impurity peaks or secondary phases.

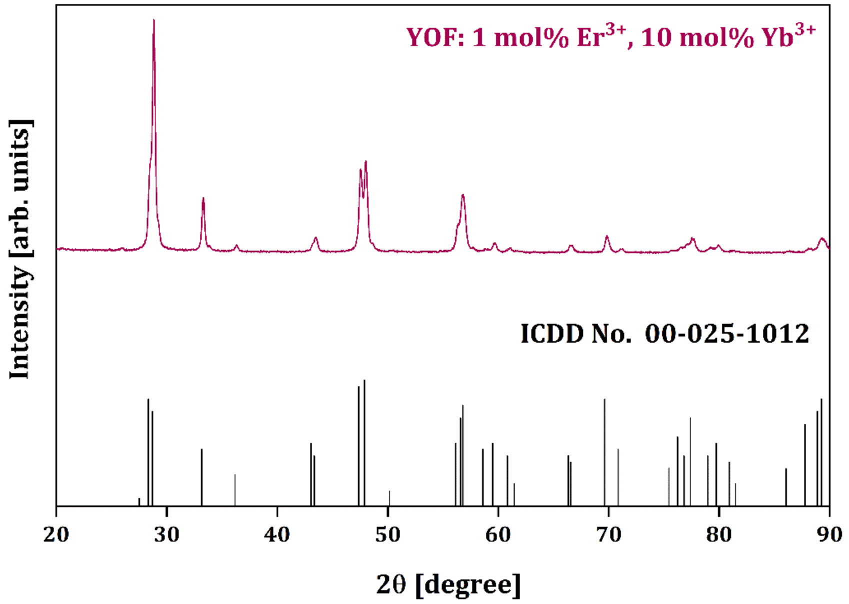

Figure S1. X-ray diffraction pattern of the YOF: Er^{3+}/Yb^{3+} nanophosphor, presented along with the ICDD card No. 00-025-1012 (crystallite size = 154 (7) Å, microstrain = 0.320 (7), and a=b= 3.797 Å, and c= 18.838 (5) Å parameters)

ML methods:

To test the LT2.0 machine learning framework, in addition to classical Boltzmann LIR and improved LIR2, the following machine learning algorithms were used:

Principal Component Analysis (PCA): an unsupervised, white-box ML technique for linear dimensionality reduction. After model training, temperature is correlated with the model output (principal components, PCs), and in controlled environments, the first principal component (PC1) typically captures 95–99% of temperature-induced spectral variation. PCA has been shown to enhance thermal resolution by 2–3× compared with conventional LIR, often achieving precision below 0.1 °C. [27]

PCA loadings were calculated using the training spectra, and only PC1 was retained for temperature estimation. The mean PC1 value at each calibration temperature defines a calibration point (s_j, T_j). For a test spectrum with PC1 value (s), the distances d_j=|s-s_j| were calculated, and the two nearest calibration points were selected. For nonzero distances, the temperature estimate was obtained by inverse-distance weighting:

$$\hat{T} = \frac{T_1/d_1 + T_2/d_2}{1/d_1 + 1/d_2},$$

where $d_1 \leq d_2$, where and T_1 and T_2 are the temperatures associated with the nearest and second-nearest calibration scores, respectively. An exact match to a calibration score returned its associated temperature. For test scores above the maximum calibration score, the implementation instead used

$$\hat{T} = T_1 + (T_1 - T_2)\frac{d_1}{d_2 - d_1}.$$

Thus, PCA provided the spectral representation, while the subsequent calibration-based calculation produced the temperature estimate.

Partial least-squares (PLS) regression: a supervised linear method that extracts latent variables as linear combinations of the input features, chosen to maximize covariance with the response variable while also maximizing variance in the predictors. Unlike principal component analysis (PCA), which decomposes the input space without knowledge of the response, PLS can be thought of as a supervised extension of PCA; the decomposition is explicitly guided by the target variable, here temperature, ensuring that the retained components are those most relevant for prediction rather than those that merely capture the most spectral variance. This makes it naturally suited to spectroscopic data, where input channels are highly collinear, and it produces a fully interpretable

white-box model in which the contribution of each wavelength to the predicted temperature is encoded in explicit, inspectable regression coefficients. [77–79]

Random Forest Regression (RFR) and *Least Squares Boosting (LSB)* are both ensemble methods based on decision trees that aggregate their outputs. In RFR, decision trees are built in parallel, each on a random subsample of the data and a random subset of features. The trees are deliberately kept independent of one another. In LSB, decision trees are built sequentially, with each new tree trained to correct the residual errors left by all the trees before it. In LS-Boost specifically, the residuals being corrected are least-squares residuals. In both cases, the final prediction is simply the average of all trees' outputs (regression). Both can be considered *gray-box* supervised models, since they provide importance scores for their inputs. However, the model's final prediction results from averaging the outputs of hundreds of decision trees, making the overall decision process opaque and preventing this method from being considered white-box. Because they were initially used for classification between known classes, one must be careful when applying regression to interpolated data. [80, 81]

Support vector regression (SVR) and *Gaussian process regression (GPR):* Both are supervised, kernel-based methods that implicitly map input features into a high-dimensional space via a kernel function, making them well-suited to high-dimensional, collinear data such as luminescent emission spectra. SVR finds the regression function by identifying a hyperplane that fits the training data within a specified tolerance margin, minimizing model complexity subject to this constraint; it produces a point prediction for each new input. GPR takes a probabilistic approach to the same problem, placing a prior distribution over possible functions and updating it with the training data to yield a full posterior distribution over predictions, providing not only a predicted value but also a

principled uncertainty estimate for each new input, which is particularly valuable in thermometry, where quantifying prediction confidence is meaningful. In terms of interpretability, neither method produces an explicit, human-readable mapping from spectral features to temperature; thus, they are gray-box models, as their probabilistic, kernel-based formulations offer some interpretability while retaining the flexibility of data-driven models. [82–84]

Artificial Neural Networks (ANN): Machine learning models learn a nonlinear mapping from input features to a target variable through a series of interconnected layers of artificial neurons, each applying a weighted linear combination of its inputs followed by a nonlinear activation function. In luminescence thermometry, the network takes the emission spectrum as input and propagates it through successive hidden layers, progressively extracting abstract representations of spectral features until a final output layer produces a predicted temperature. The weights of the network are optimized during training by minimizing the difference between predicted and true temperatures across the training dataset via backpropagation, making ANNs fully supervised. In terms of interpretability, ANNs are considered ***black-box*** because their distributed, nonlinear learned representations are fundamentally opaque. There is no straightforward way for humans to trace how a particular emission spectrum leads to a predicted temperature. [85–87]

Table S1. Fitting and hyperparameter settings for the ML methods used.

**Fitting and hyperparameters**	
**LIR ($ln(LIR)=C_0+C_1*10^3/T$)**	
C_0	+1.5008$_{\text{VISIBLE}}$ | -0.4181$_{\text{NIR}}$
C_1	-0.6470 $_{\text{VISIBLE}}$ | -0.2798 $_{\text{NIR}}$

**LIR2(*$ln(LIR)=C_0+C_1*10^3/T+ C_2*10^6/T^2$*)**	
C_0	+1.8343 VISIBLE | -0.1487 NIR
C_1	-0.8925 VISIBLE | -0.4782 NIR
C_2	+0.0445 VISIBLE | +0.0360 NIR
**PCA (using *pca()* MATLAB function)**	
Algorithm	Singular value decomposition - SVD
Number of principal components used for temperature estimation	1 (PC1)
**PLS (using *plsregress()* MATLAB function)**	
Algorithm	Statistically Inspired Modification of PLS - SIMPLS
Number of components	3 **OThO*
**RF (using *TreeBagger()* MATLAB function)**	
Algorithm	regression
Number of trees	1000
Minimum number of leaf node observations	32
InBagFraction	0.8
Number of predictor variables for each decision split	54
**LS boost (using *fitrensemble()* MATLAB function)**	
Method	LSBoost
Number of Learning Cycles	494
Learning Rate	0.71653
Maximum Number of decision Splits	770
**SVR (using *fitrsvm()* MATLAB function)**	
Method	Sequential Minimal Optimization (SMO)
Kernel Function	Linear
Box Constraint	21.101_{MAX} ; 50.38_{AREA} ; 994.9_{ZSCORE} **OThO*
Kernel Scale	0.021918_{MAX} ; 0.0023826_{AREA} ; 11_{ZSCORE} **OThO*
Epsilon	0.1785_{MAX} ; 0.059528_{AREA} ; 0.074857_{ZSCORE} **OThO*
**GPR (using *fitrgp()* MATLAB function)**	

Method	Exact marginal log-likelihood maximization
Kernel Function	Squared exponential
**ANN (using *fitrnet()* MATLAB function)**	
Architecture	Two hidden layers, with candidate sizes of {5, 10, 15} neurons in the first layer and {2, 4, 6, 8, 10, 12} neurons in the second layer, followed by one output neuron. The architecture was selected by minimizing training-set RMSE.
Activation functions (hidden nodes)	hyperbolic tangent sigmoid
Activation functions (output node)	linear

Hyperparameter selection:

Hyperparameter optimization was performed separately from the GUI used for subsequent model fitting and evaluation. RF, LSB, SVR, and GPR were optimized using MATLAB's Bayesian optimization routines with the expected-improvement-plus acquisition function and the default five-fold cross-validation within the training dataset. These internal folds were not grouped by temperature and were distinct from the held-out interpolation-temperature test used to evaluate predictive performance.

The RF search varied the number of trees, minimum leaf size, and number of predictors sampled at each split. The LSB search varied the number of learning cycles, learning rate, and maximum number of tree splits. For SVR, the search used the optimizable-variable configuration returned by MATLAB's hyperparameters function. For GPR, the search varied Sigma and KernelScale with a squared-exponential kernel. The supplied RF, LSB, and GPR optimization procedures specified a maximum of 30 objective evaluations.

ANN architecture selection used a grid search over 18 two-hidden-layer configurations: 5, 10, or 15 neurons in the first hidden layer and 2, 4, 6, 8, 10, or 12 neurons in the second.

Each configuration was fitted to the training dataset, and the architecture yielding the lowest training-set RMSE was selected. No separate validation subset or cross-validation was used for architecture selection.

Synthetic-background generation and training-set augmentation:

Synthetic backgrounds were generated within the selected spectral window after subtracting the minimum intensity from each spectrum. Let $I_0(\lambda) = I(\lambda) - \min_{\lambda} I(\lambda)$ denote the resulting spectrum, with wavelength limits $\lambda_{\min}$ and $\lambda_{\max}$. The dimensionless background profiles were defined as

$$b_{\text{lin}}(\lambda) = \frac{\lambda - \lambda_{\min}}{\lambda_{\max} - \lambda_{\min}}, \quad b_{\text{R}}(\lambda) = \frac{\lambda^{-4} - \lambda_{\max}^{-4}}{\lambda_{\min}^{-4} - \lambda_{\max}^{-4}}.$$

Both profiles range from zero to unity. A perturbed spectrum was generated as

$$I_{\text{BG}}(\lambda) = I_0(\lambda) + \frac{p}{100} \max_{\lambda}[I_0(\lambda)] \; b(\lambda),$$

where p is the prescribed background level expressed as a percentage. Thus, the maximum added background was scaled to the peak intensity of each individual spectrum. For the VIS analysis, the nonzero levels were 0.1, 0.5, 1.0, 1.5, and 2.0%; the unperturbed spectra provided the 0% reference. No random amplitude sampling was used.

Augmentation was performed separately for each background shape. The original 800 training spectra were retained, and one perturbed copy of every training spectrum was added at each of the five nonzero levels, yielding 4000 additional spectra and an augmented training set of 4800 spectra. Each copy retained the temperature label of its

source spectrum. MAX, AREA, or ZSCORE normalization was applied after background addition. For each method and normalization, separate models were fitted to the original and augmented training sets and subsequently evaluated on the same reserved test spectra at every prescribed background level. Test spectra were not included in either training set. This matched-background protocol evaluates tolerance to the specified perturbations rather than general resistance to unrepresented experimental interference.

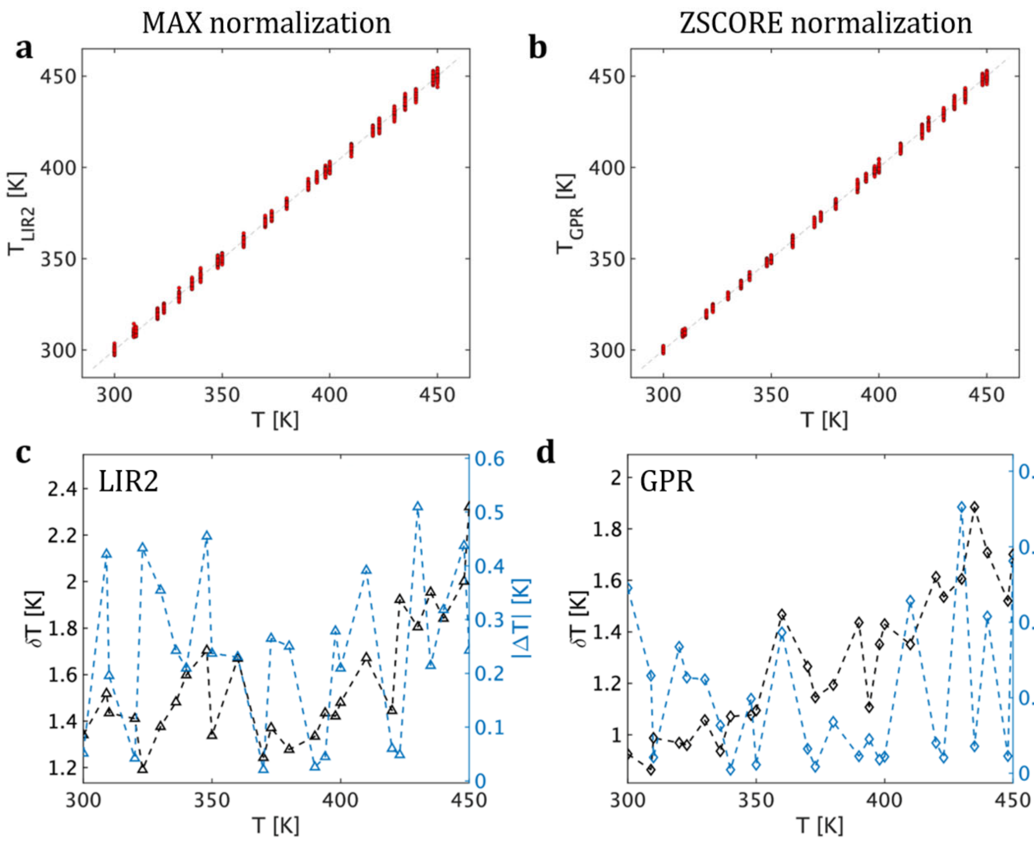

Figure S2. VIS spectral range. Predicted vs. stabilized temperatures for **a.** LIR2 and **b.** GPR, and calculated accuracy (diamonds) and precision (circles) for **c.** LIR2 and **d.** GPR.

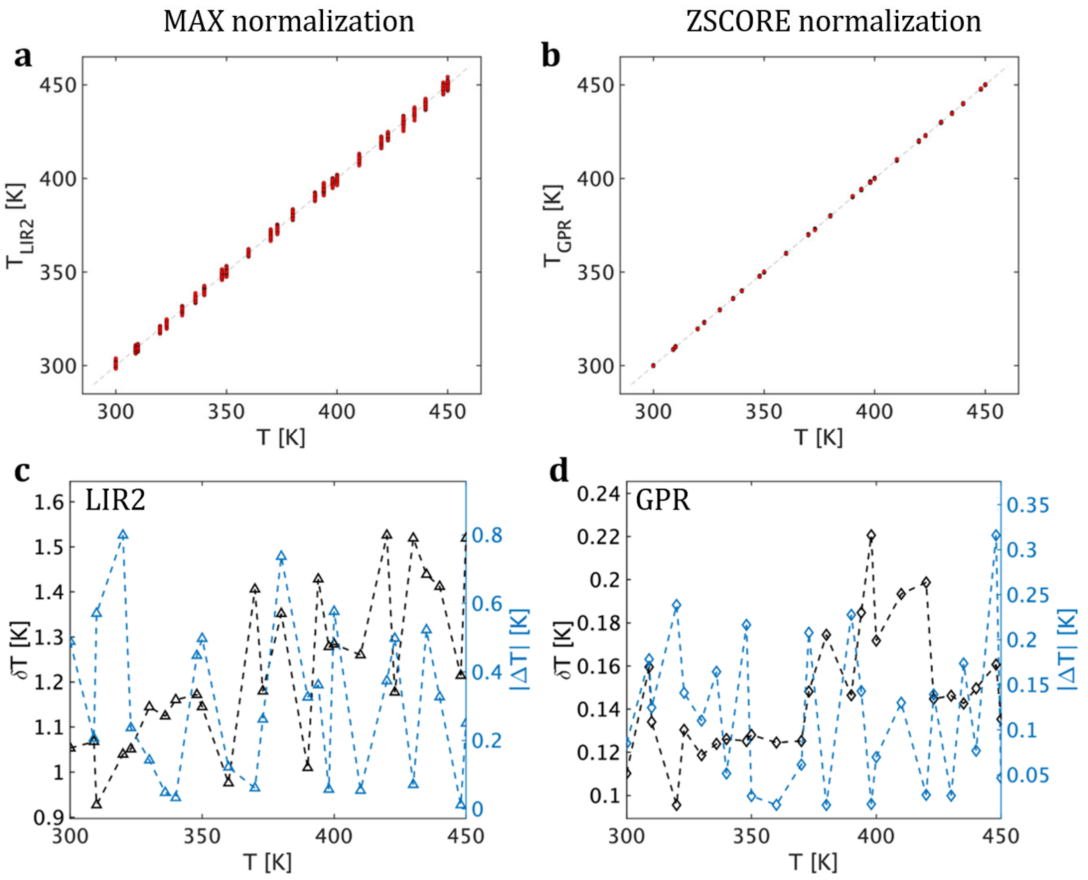

Figure S3. NIR spectral range. Predicted vs. stabilized temperatures for **a.** LIR2 and **b.** GPR, and calculated accuracy (diamonds) and precision (circles) for **c.** LIR2 and **d.** GPR.

Table S2. Thermometric performance at each stabilized temperature. Accuracy, ΔT, and precision, δT, obtained from VIS UC and NIR downshifting emission spectra using conventional LIR-based thermometry and full-spectrum ML regression. Results are shown for maximum, area, and ZSCORE spectral normalization. Lower ΔT and δT values indicate better accuracy and precision, respectively.

VIS spectral range, thermometric performance
absolute bias, ΔT [K] (accuracy) and prediction spread, δT [K] (precision)

Norm.→	MAX		AREA		ZSCORE	
**Method**	ΔT	δT	ΔT	δT	ΔT	δT
**LIR**	0.85	1.58	0.85	1.58	—	—
**LIR2**	**0.24**	**1.56**	**0.24**	**1.56**	—	—
**PCA**	0.37	3.03	0.66	3.07	0.19	1.29
**PLS**	0.64	1.55	1.23	2.00	0.20	**1.25**
**RF**	0.58	2.39	0.96	2.72	0.73	2.02
**LSB**	0.49	3.15	0.70	4.15	0.54	3.77
**SVR**	0.57	1.77	0.98	2.37	0.20	1.28
**GPR**	0.20	1.30	0.21	1.29	**0.20**	1.28
**ANN**	0.33	1.43	0.57	1.41	0.51	1.60

NIR spectral range, thermometric performance
absolute bias, ΔT [K] (accuracy) and prediction spread, δT [K] (precision)

Norm.→	MAX		AREA		ZSCORE	
**Method**	ΔT	δT	ΔT	δT	ΔT	δT
**LIR**	1.50	1.26	1.50	1.26	—	—
**LIR2**	**0.31**	**1.23**	**0.31**	**1.23**	—	—
**PCA**	0.35	0.31	0.27	0.66	0.21	**0.12**
**PLS**	0.23	0.28	0.29	0.68	0.24	0.14
**RF**	0.67	0.47	0.59	0.32	0.74	0.16
**LSB**	0.82	0.90	0.91	0.91	0.79	0.69
**SVR**	0.11	0.15	0.10	0.20	0.21	**0.14**
**GPR**	0.12	0.14	0.09	**0.13**	0.12	0.15
**ANN**	0.12	0.15	0.16	0.15	**0.08**	0.15

Results shown for each stabilized temperature. Lower *ΔT* and δT indicate better performance.

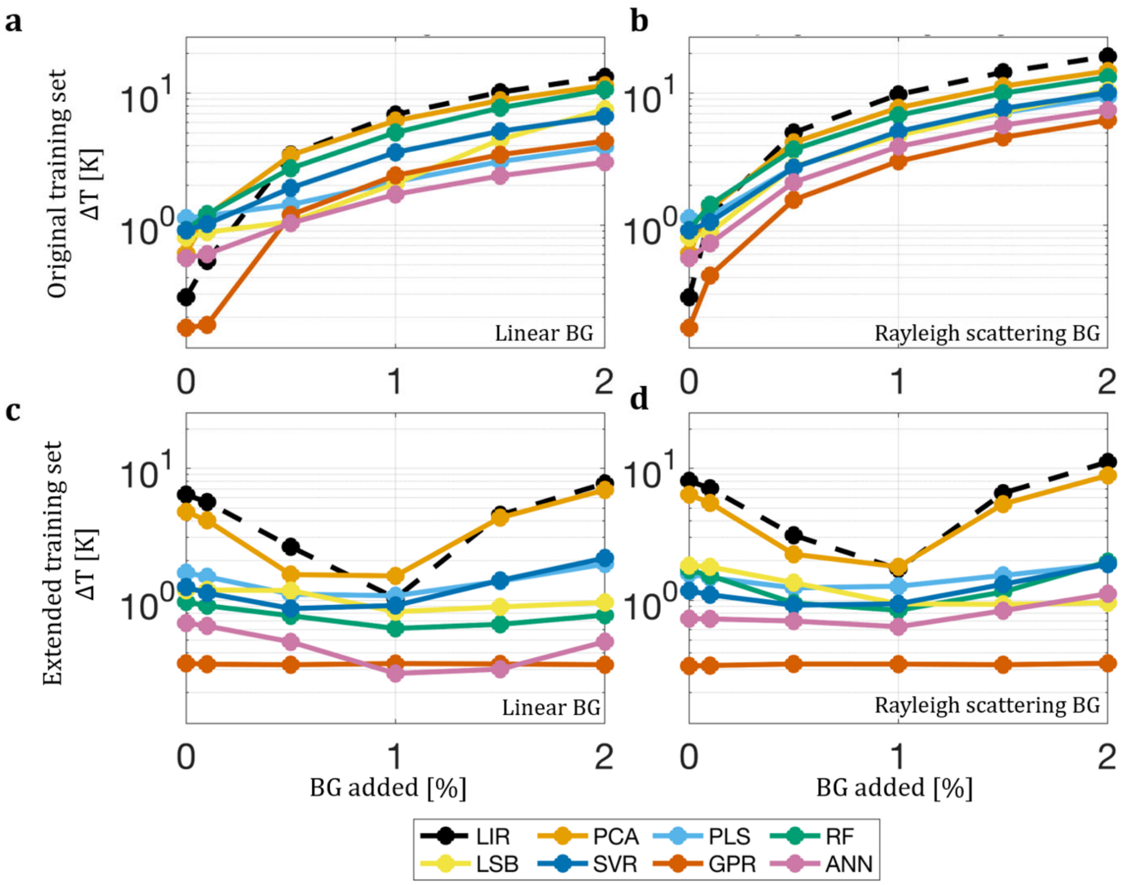

Figure S4. VIS spectral range, AREA normalization. Absolute bias, ΔT (accuracy) for all eight methods, trained on the original spectra: **a.** linear, **b.** Rayleigh-scattering background (BG), and on the extended training set: **c.** linear, **d.** Rayleigh-scattering background (BG).

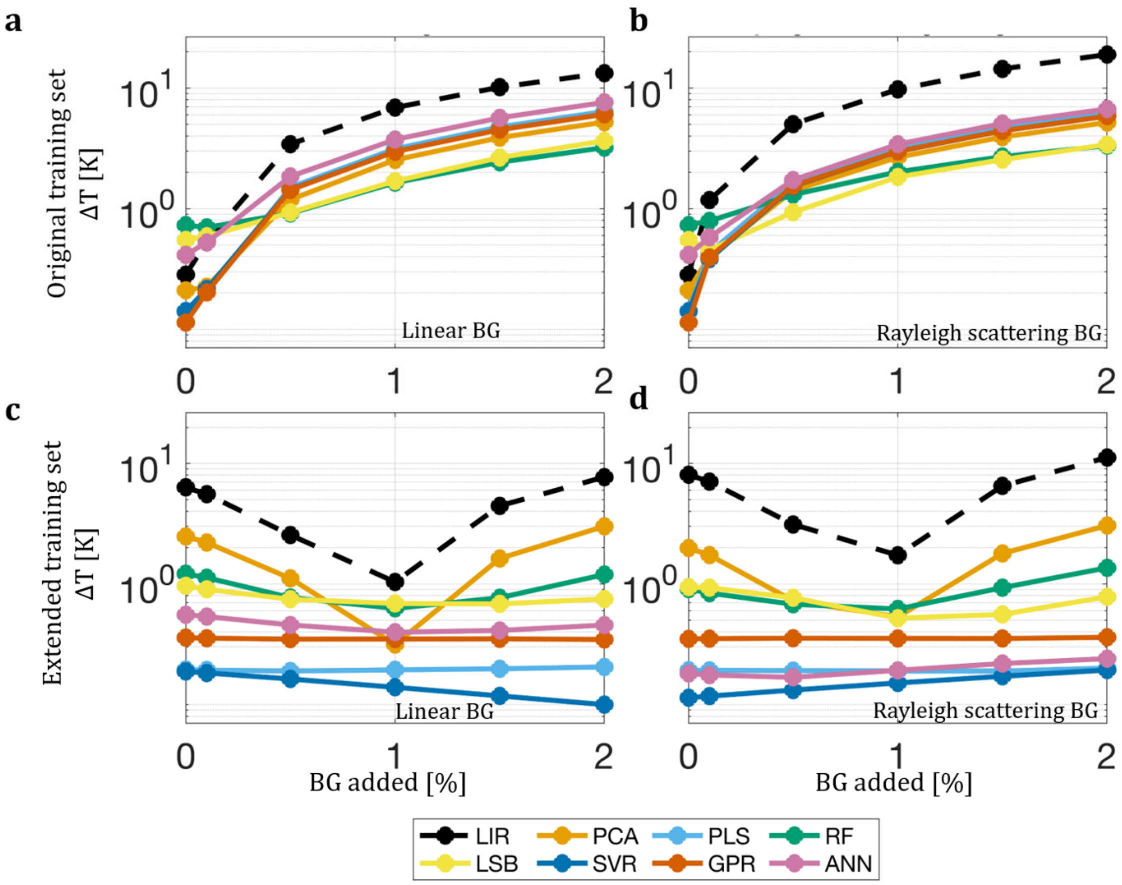

Figure S5. VIS spectral range, ZSCORE normalization. Absolute bias, ΔT (accuracy) for all eight methods, trained on the original spectra: **a.** linear, **b.** Rayleigh-scattering background (BG), and on the extended training set: **c.** linear, **d.** Rayleigh-scattering background (BG).

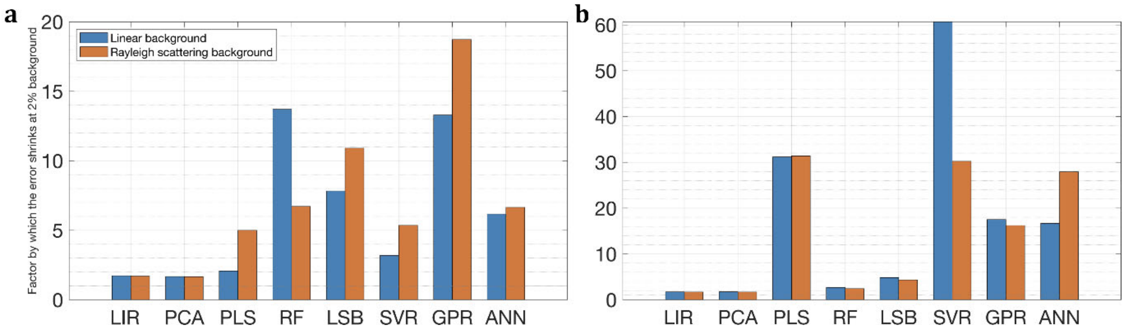

Figure S6. Relative improvement in the absolute bias, ΔT (accuracy) at the highest background level (2%) on the extended training set: **a.** AREA normalization; **b.** ZSCORE normalization.

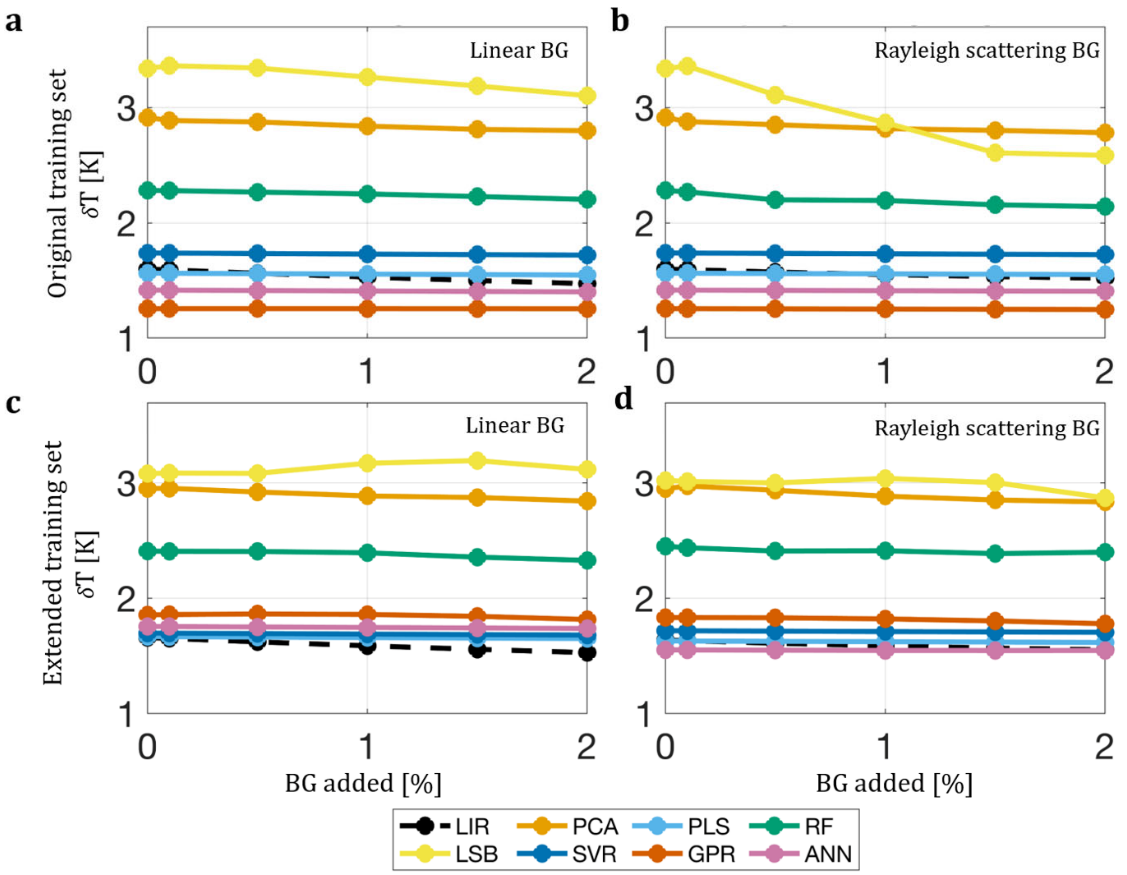

Figure S7. MAX normalization. Prediction spread, δT (precision) for all eight methods, trained on the original spectra: **a.** linear, **b.** Rayleigh-scattering background (BG), and on the extended training set: **c.** linear, **d.** Rayleigh-scattering background (BG).

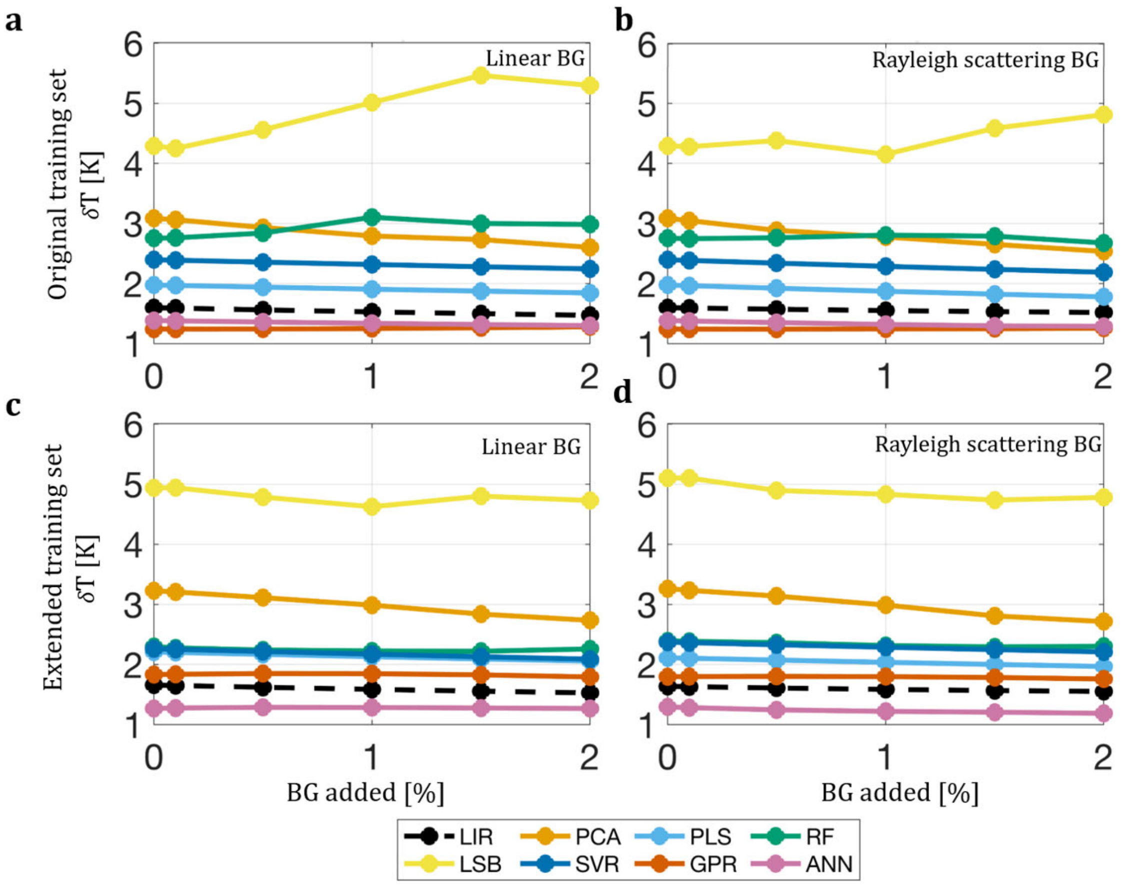

Figure S8. AREA normalization. Prediction spread, δT (precision) for all eight methods, trained on the original spectra: **a.** linear, **b.** Rayleigh-scattering background (BG), and on the extended training set: **c.** linear, **d.** Rayleigh-scattering background (BG).

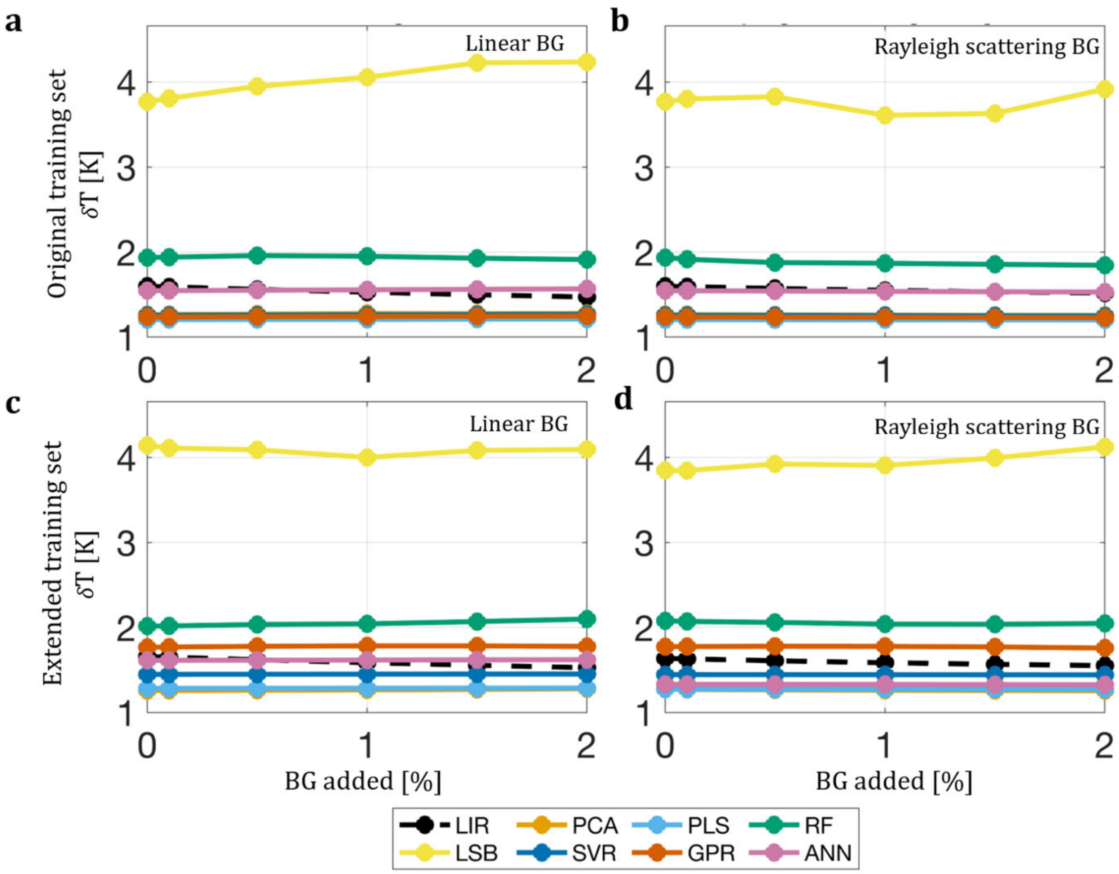

Figure S9. ZSCORE normalization. Prediction spread, δT (precision) for all eight methods, trained on the original spectra: **a.** linear, **b.** Rayleigh-scattering background (BG), and on the extended training set: **c.** linear, **d.** Rayleigh-scattering background (BG).

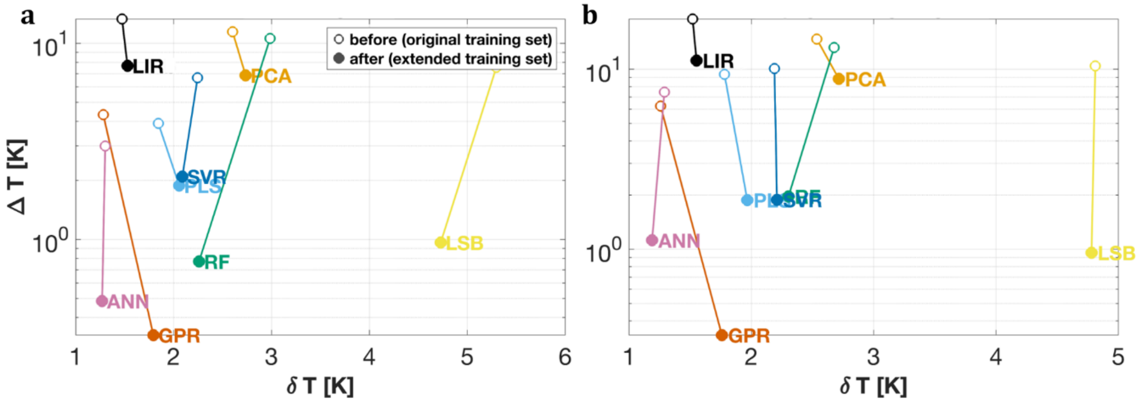

Figure S10. Accuracy (ΔT, vertical axis, log scale) and prediction spread, δT (precision; horizontal axis), at 2%. **a.** linear, and **b.** Rayleigh-scattering background (AREA normalization, lower is better).

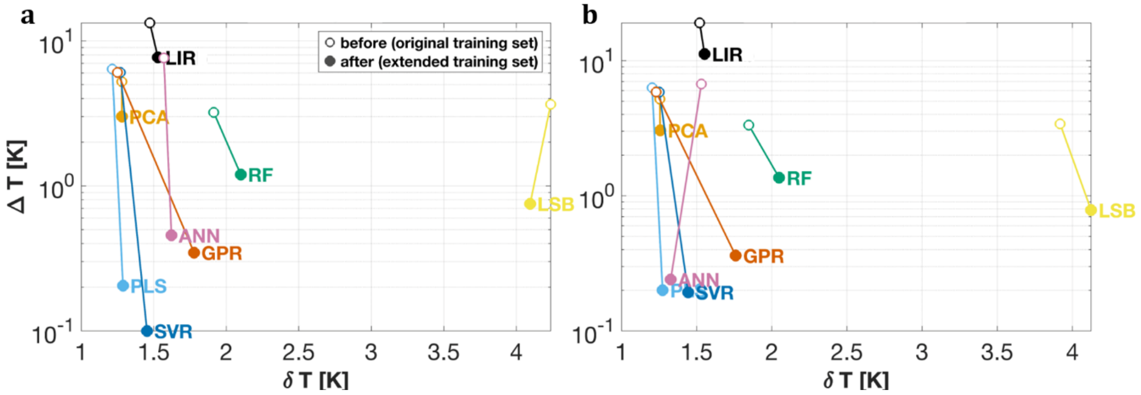

Figure S11. Absolute bias, ΔT (accuracy; vertical axis, log scale) and prediction spread, δT (precision; horizontal axis), at 2%. **a.** linear, and **b.** Rayleigh-scattering background (ZSCORE normalization, lower is better).

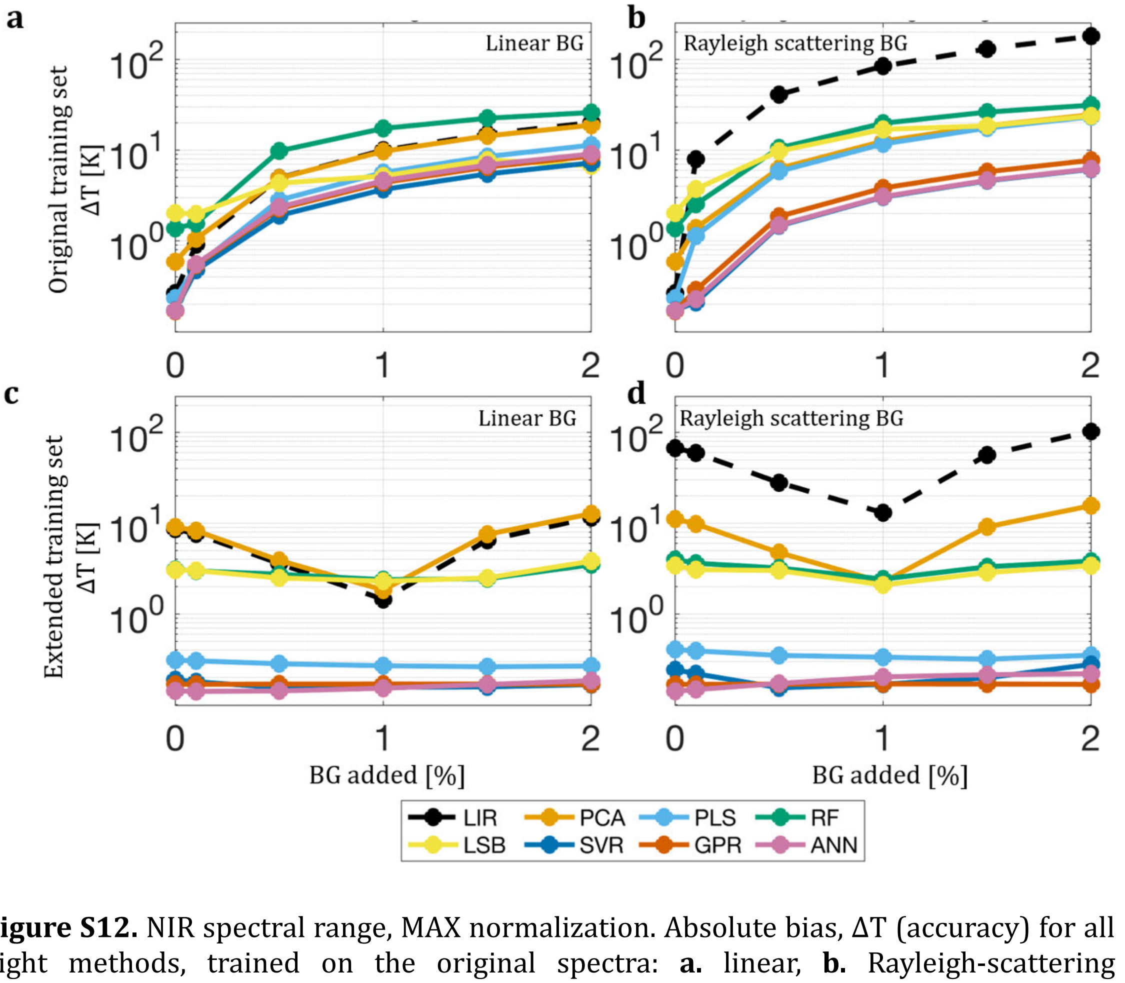

Figure S12. NIR spectral range, MAX normalization. Absolute bias, ΔT (accuracy) for all eight methods, trained on the original spectra: **a.** linear, **b.** Rayleigh-scattering background (BG), and on the extended training set: **c.** linear, **d.** Rayleigh-scattering background (BG).

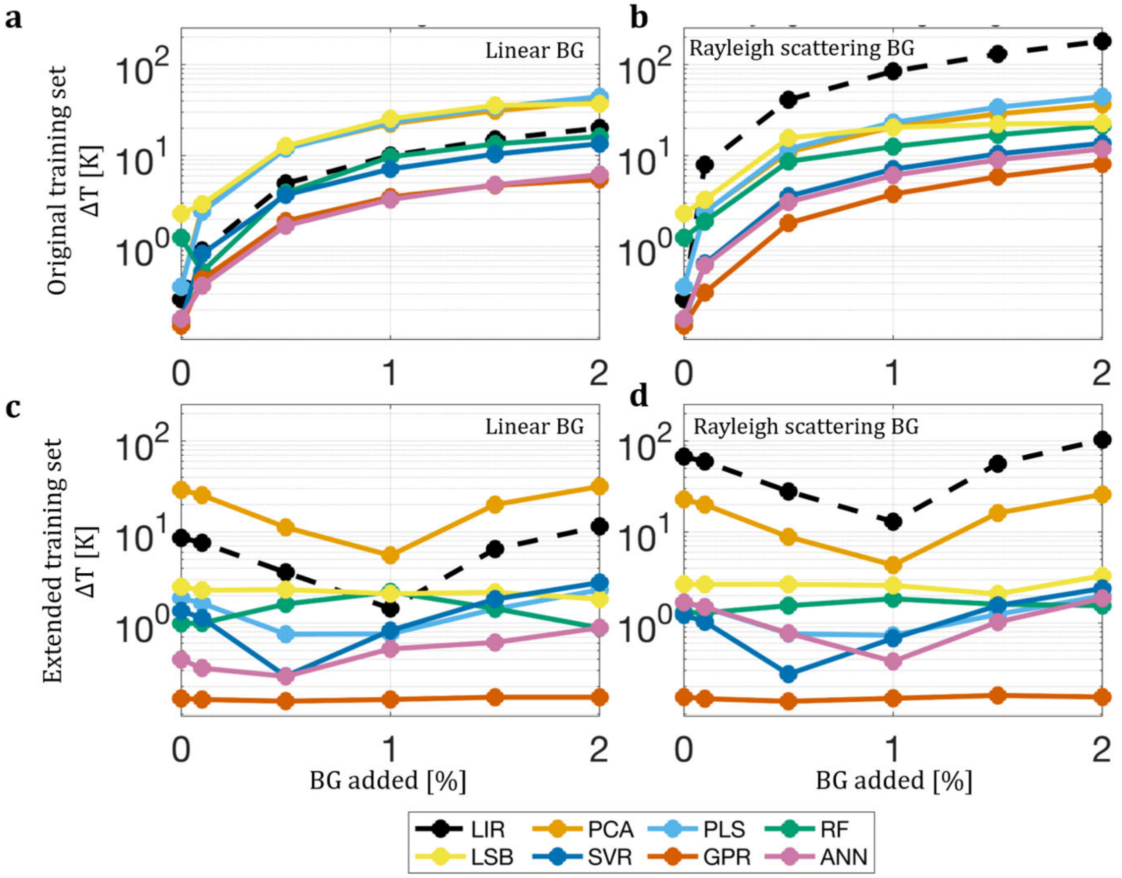

Figure S13. NIR spectral range, AREA normalization. Absolute bias, ΔT (accuracy) for all eight methods, trained on the original spectra: **a.** linear, **b.** Rayleigh-scattering background (BG), and on the extended training set: **c.** linear, **d.** Rayleigh-scattering background (BG).

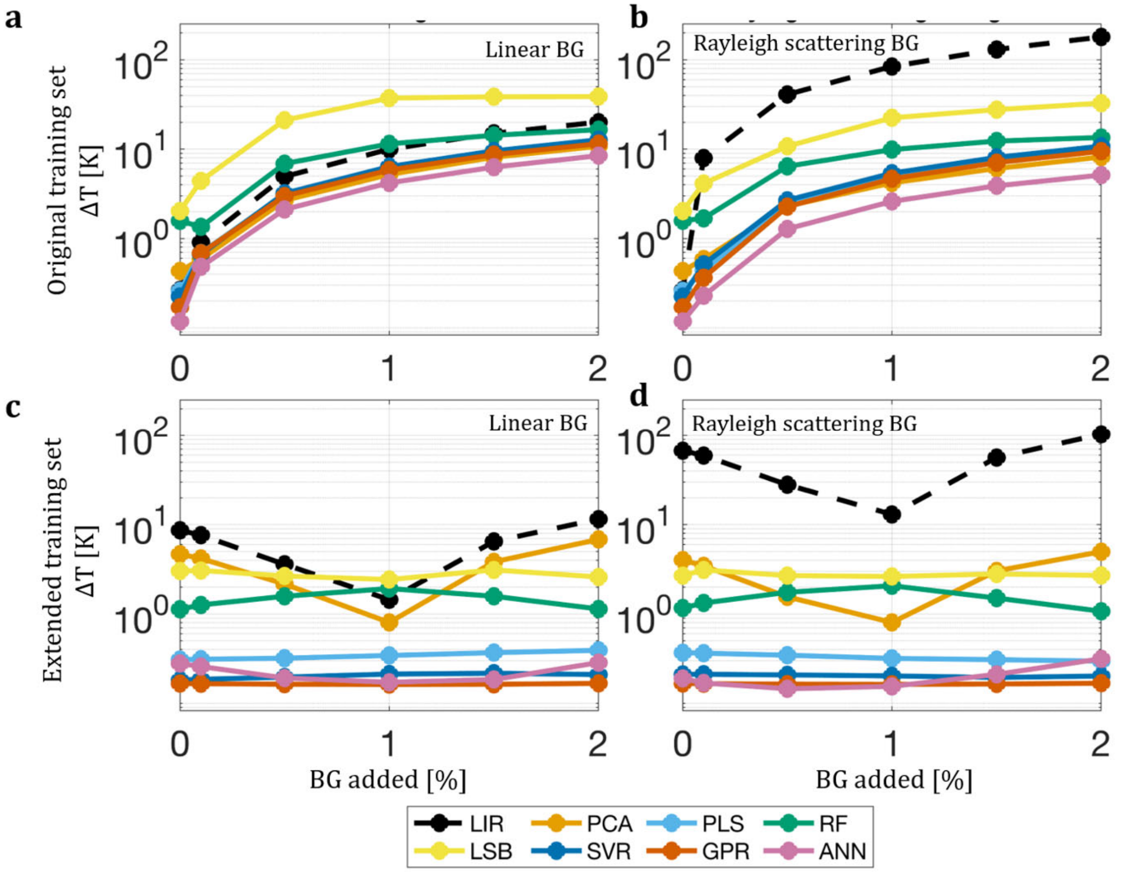

Figure S14. NIR spectral range, ZSCORE normalization. Absolute bias, ΔT (accuracy) for all eight methods, trained on the original spectra: **a.** linear, **b.** Rayleigh-scattering background (BG), and on the extended training set: **c.** linear, **d.** Rayleigh-scattering background (BG).
